# Immunoglobulin gene rearrangements in Chinese and Italian patients with chronic lymphocytic leukemia

**DOI:** 10.18632/oncotarget.7819

**Published:** 2016-03-01

**Authors:** Marilisa Marinelli, Caterina Ilari, Yi Xia, Ilaria Del Giudice, Luciana Cafforio, Irene Della Starza, Sara Raponi, Paola Mariglia, Silvia Bonina, Zhen Yu, Wenjuan Yang, Lugui Qiu, Thomas Chan, Alfonso Piciocchi, Yok-Lam Kwong, Eric Tse, Jianyong Li, Anna Guarini, Wei Xu, Robin Foà

**Affiliations:** ^1^ Department of Cellular Biotechnologies and Hematology, “Sapienza” University, Rome, Italy; ^2^ Department of Hematology, The First Affiliated Hospital of Nanjing Medical University, Jiangsu Province Hospital, Nanjing, China; ^3^ Department of Lymphoma & Myeloma Institute of Hematology, CAMS & PUMC, Tianjin, China; ^4^ Department of Medicine, The University of Hong Kong, Queen Mary Hospital, Hong Kong; ^5^ GIMEMA Data Center, GIMEMA Foundation, Rome, Italy

**Keywords:** chronic lymphocytic leukemia, IGHV, stereotyped receptors, Chinese CLL, Italian CLL

## Abstract

Chronic lymphocytic leukemia (CLL) is the most common type of leukemia in the Western world, whereas in Asia the incidence is about 10 times lower. The basis for this ethnic and geographic variation is currently unknown. The aim of this study was to characterize IGHVDJ rearrangements and stereotype of the HCDR3 region in a series of 623 Chinese CLL, in order to identify possible differences in immunoglobulin gene usage and their potential pathogenetic implications. Chinese CLL were compared to 789 Italian CLL. Chinese patients showed a higher proportion of mutated IGHV and a more frequent usage of IGHV3-7, IGHV3-74, IGHV4-39 and IGHV4-59 genes. A significantly lower usage of IGHV1-69 and IGHV1-2 was documented, with comparable IGHV3-21 frequency (3% Chinese vs 3.8% Italian CLL). The proportion of known stereotyped receptors was significantly lower in Chinese (19.7%) than in Italian CLL (25.8%), despite a significantly higher frequency of subset #8 (*p*= 0.0001). Moreover, new paired clusters were identified among Chinese cases. Overall, these data support a potential different antigenic exposure between Eastern and Western CLL.

## INTRODUCTION

Chronic lymphocytic leukemia (CLL) is characterized by a variable clinical course, driven by the immunogenetic and molecular heterogeneity of the disease [1-5].

The heterogeneity of CLL is also reflected by the different incidence in populations with diverse ethnic and geographic distribution. CLL is the most common leukemia among adults in the Western hemisphere (~30% of all leukemias), but is rare in Asian countries [6, 7]. The age-adjusted incidence rate (AAIR) is about 4.4 new cases x 100.000 individuals/year in the US (2007-2011) [8]. In contrast, the AAIR in Asians is about 10 times lower, with 0.2-0.3 new cases x 100.000 individuals/year [8, 9]. The reasons for this epidemiologic heterogeneity remain to be defined. The risk of developing CLL does not change among Asians residing in the US and in their descendants [10, 11]. Therefore, the genetic background is important in determining the risk of the disease, beyond potential environmental factors.

There are some data on the clinico-biologic features of Asian CLL [12-14]. The prognostic impact of CD38 [15], ZAP-70 [16], genetic lesions evaluated by FISH [17, 18] and of other biologic markers such as CLLU1 [19], lipoprotein lipase [20], serum thymidine-kinase [21] seems confirmed also in Asians CLL. On the contrary, a different clinical presentation, a younger age at diagnosis and a worse prognosis have been reported [22]. Furthermore, some differences between Chinese and European CLL in the incidence and prognostic impact of mutations in driver genes (*SF3B1, NOTCH1, MYD88, BIRC3, TP53*) have been suggested [23].

To date, little is known on the IGHV gene representation and mutations in Asian compared to Caucasian CLL. Indeed, some differences have so far been reported only in small cohorts of patients [24-27]. The B-cell receptor (BCR) repertoire in CLL is influenced by the genetic susceptibility and/or by the existence of a promoting pressure derived from different antigenic elements. The potential role of antigens in the pathogenesis of CLL is further supported by the presence of the non-stochastic pairing of IGHV, IGKV and IGLV genes, which leads to nearly identical BCR sequences of heavy chain complementarity determining region 3 (HCDR3) region, described as “stereotyped” BCR, in about 30% of Caucasian CLL [28].

In this study, we investigated IGHV gene usage, mutational status and HCDR3 in the largest series of Chinese CLL so far reported, and compared them to those present in Italian CLL. Despite general analogous features, Chinese and Italian CLL showed significant differences in IGHVDJ gene usage, mutations and stereotypy. These findings raise the hypothesis that diverse specific antigens are implicated in the pathogenesis of Asian CLL.

## RESULTS

### Italian CLL: IGHV gene usage, mutation analysis and HCDR3 features

A total of 792 IGHVDJ productive rearrangements from 789 Italian CLL cases were evaluated; 2 cases carried double and triple in-frame rearrangements, respectively. Using the 2% cut-off to discriminate between mutated and unmutated rearrangements, 387 (49%) of the 792 sequences analyzed had more than 2% difference from the most similar germline gene and were classified as mutated with a median of 7.4% (range 2-17%) mutations/case The remaining 405 (51%) were considered as unmutated, of which 278 (68%) displayed a 100% sequence identity to the corresponding germline IGHV gene sequences (Supplementary Table S1A). The IGHVDJ subgroup and gene usage were for the most part comparable to those reported in previous studies [28-31], showing no relevant differences in the frequency of the most representative genes. As expected, the analysis revealed that the most common IGHV subgroup was IGHV3 (364; 46.0%), followed by IGHV1 (199; 25.1%) and IGHV4 (163; 20.6 %) (Supplementary Table S1B).

When we examined the IGHV genes, the most frequently encountered were IGHV1-69 (110; 13.9%), IGHV4-34 (77; 9.7%), IGHV3-23 (51; 6.4%), IGHV3-30 (46; 5.8%), IGHV1-2 (44; 5.6%), IGHV3-7 (42; 5.3%), IGHV3-11 (31; 3.9%), IGHV3-21 (30; 3.8%), IGHV3-48 (28; 3.5%), IGHV4-39 (27; 3.4%), IGHV3-33 (27; 3.4%); collectively, these genes accounted for 65% of the cases, in line with the reported features of Caucasian CLL (Supplementary Table S1B). In our cohort, we also confirmed the known low frequency of the IGHV3-21 gene in CLL patients of Mediterranean origin, with one third of cases being mutated [29].

As expected, the IGVH1-69 gene was over-represented in the unmutated group (*p*=0.0001), whilst IGHV4-34 (*p*=0.0001), IGHV3-7 (*p*=0.0001) and IGHV3-23 (*p*=0.0001) were over-represented in the mutated group.

Among the 792 sequences, IGHD genes were identified in 774 cases and IGHD3 was the most frequently represented (337; 43.5%) followed by IGHD2 (135; 17.4%) and IGHD6 (107; 13.8%). The IGHD3-3 (133; 17.2%) gene was the most frequently used followed by IGHD3-22 (82; 10.6%) (Supplementary Table S1C).

With regard to the IGHJ gene usage, the most frequent subgroup was IGHJ4 (332; 41.9%), followed by IGHJ6 (264; 33.3%) and IGHJ5 (89; 11.2%) (Supplementary Table S1D). Moreover, IGHJ4 recurred in mutated sequences (*p*= 0.0001), whilst IGHJ6 was recurrent in unmutated rearrangements (*p*= 0.0001).

We also confirmed the presence of specific IGHV, IGHD and IGHJ gene combinations, in particular, for the IGHV1-69 gene the strongly biased association with IGHD3-3 (32/110; 29%) (*p*= 0.0005) and IGHJ6 (63/110; 57%) segments (*p*= 0.0001).

The median length of HCDR3 was 19 amino acids (range, 7-35). Unmutated sequences had significantly longer HCDR3s than mutated sequences: median length 22 versus 17 amino acids (*p*= 0.0001).

Cluster analysis of the HCDR3 region allowed to recognize 204 of 792 cases (25.8%) with stereotyped BCRs (Supplementary Table S1E–S1F) belonging to 72 different subsets. We confirmed a significantly higher frequency of unmutated (153/405; 37.7%) than mutated (51/387; 13.2%) stereotyped sequences (*p*= 0.0001). Of the major stereotyped subsets of Caucasian CLL, the most frequent in the present Italian series were subset #1 (20.9%), #4 (11.3%) and #2 (10.4%).

The most represented IGHV genes in stereotyped HCDR3 were: IGHV1-69 (#3, #5, #6, #7), IGHV4-34 (#4) and IGHV3-21 (#2).

Moreover, we identified 10 (4.9%) sequences that fell into 5 groups with identical HCDR3 and thus were defined as paired clusters.

### Chinese CLL: IGHV gene usage, mutation analysis and HCDR3 features

A total of 631 productive IGHVDJ rearrangements were sequenced from the cohort of 623 Chinese CLL patients; 8 (1.3%) cases showed biallelic IGHVDJ rearrangements. A total of 415 of the 631 sequences analyzed (66%) were defined as mutated, with a median of 7% (range 2-22%) mutations/case, whereas 216 (34%) sequences had unmutated IGHV genes, with 121 (56%) in 100% germline configuration (Supplementary Table S2A).

Analysis of the IGHV subgroups showed a higher usage of IGHV3 (295; 46.8%) followed by IGHV4 (181; 28.7%) and IGHV1 (98; 15.5%) (Supplementary Table S2B). The most frequent IGHV genes were IGHV4-34 (70; 11.1%), IGHV3-23 (53; 8.4%), IGHV3-7 (51; 8.1%), IGHV4-39 (43; 6.8%), IGHV1-69 (36; 5.7%), IGHV4-59 (30; 4.8%), IGHV3-30 (27; 4.3%), IGHV3-74 (23; 3.7%), IGHV1-3 (23; 3.7%) and IGHV3-48 (22; 3.5%). Overall, these genes were found in 60% of the analyzed cases. Cumulative results are summarized in Supplementary Table S2B. The known biased usage was found, with IGVH1-69 (*p*= 0.0001) gene over-represented in the unmutated group, and IGHV4-34 (*p*= 0.0001) and IGHV3-7 (*p*= 0.003) in the mutated group. IGHV3-21 represented the eleventh group in frequency (19; 3%), with two thirds being mutated (mutated, n=13; unmutated, n=6).

IGHD genes were identified in 622 of 631 productive rearrangements. Overall, the most frequently used subgroup was IGHD3 (239; 38.4%), followed by IGHD6 (122; 19.6%) and IGHD2 (91; 14.6%) (Supplementary Table S2C). The most prevalent IGHD genes were IGHD3-10 (74; 11.9%) and IGHD3-22 (59; 9.5%).

Regarding IGHJ gene usage, among Chinese patients there was an over-representation of the IGHJ4 (289; 45.8%) and IGHJ6 (161; 25.5%) genes (Supplementary Table S2D). In addition, IGHJ4 recurred in mutated rearrangements (*p*= 0.0001), whilst IGHJ6 was recurrent in unmutated rearrangements (*p*= 0.0028). Focusing on IGHVDJ rearrangements, we identified a biased association of IGHV1-69 with IGHD3-3 and JH6 gene, similarly to Caucasian CLL, although a significant correlation was observed only with IGHJ6 (*p*=0.016).

The HCDR3 median length was 17 amino acids (range, 7-35). Significantly longer HCDR3s were observed in unmutated versus mutated sequences (median lengths, 21 versus 16 amino acids; *p*= 0.0001). The frequency of a stereotyped HCDR3 was significantly higher among unmutated (72/216; 33.3%) than mutated (52/415; 12.5%) sequences (*p*= 0.0001). We identified 124 stereotyped HCDR3 out of 631 cases (19.7%), belonging to 48 different subsets, of which 16 (12.9%) have not been reported previously and were named as novel clusters (see below). The major stereotyped subsets of Caucasian CLL collectively represent a substantial proportion of stereotyped sequences also in the Chinese series, with a preferential recurrence of subset #8 (27.3%), #1 (21.2%), #4 (16.7%), while the subset #2 (1.5%) appeared to be less represented (Supplementary Table S2E).

By comparing the IGHV usage of stereotyped sequences with those not belonging to any cluster, the IGHV1 (*p*= 0.03) and IGHV4 (*p*= 0.0004) subgroups were the most frequently utilized in stereotyped sequences, whereas IGVH3 was preferentially employed by non-homologous sequences (*p*= 0.0001). Indeed, the most frequent stereotyped IGHV genes were: IGHV4-34 (#4) IGHV4-39 (#8) and finally IGHV1-69 (#3, #5, #6, #7).

Regarding the 16 novel clusters (corresponding to 33 sequences), two were potential new subsets (composed of 3 Chinese CLL and 2 Chinese plus 2 Italian CLL, respectively) and 14 were paired clusters (all Chinese), as shown in Supplementary Table S2F. In contrast to common subsets, the majority of novel clusters were mutated (19/33 sequences; 57.5%). Of interest, 15 of 19 novel mutated sequences expressed genes from the IGHV3 subgroup, of which IGHV3-23 (8/33; 24.2%) was the most represented gene.

### Comparison between Italian and Chinese CLL reveals differences in IGHV mutational status and gene repertoire

Comparing the two series, Chinese CLL showed a higher proportion of cases with mutated IGHV than Italian CLL. Such difference appeared to be statistically significant - mutated IGHV 66% and unmutated IGHV 34% vs mutated IGHV 49% and unmutated IGHV 51%, respectively (*p*= 0.0001) - and remained significant also after exclusion of pre-treated cases (*p*= 0.0001).

**Figure 1:**
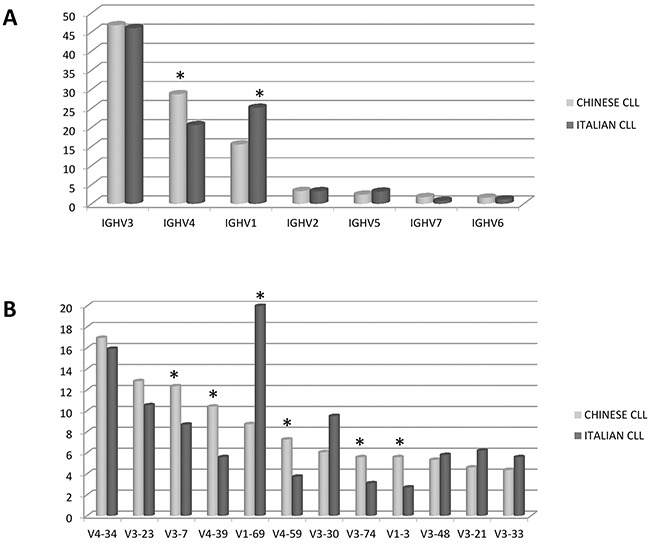
Comparison between Chinese and Italian CLL **A.** Frequency of the IGHV subgroups. **B.** Frequency of the IGHV genes. * marks the significant differences (p values in the text). Data are represented as percentage.

Whilst the IGHV3 subgroup was predominantly expressed both in Chinese and Italian patients, there was an inversion between IGHV1 and IGHV4 frequency. In fact, the frequency of IGVH1 in Chinese CLL resulted lower (*p*= 0.0001), while the IGHV4 subgroup was significantly higher (*p*= 0.0004) (Figure 1A).

In line with these results, the IGHV gene usage highlighted differences in the 10 most frequent genes of Italian and Chinese CLL. Overall, the 10 most frequent IGHV genes employed by Italian CLL represented 50% of the IGHV genes used by the Chinese CLL, showing a significant difference (*p*= 0.0001). In fact, IGHV1-69 (*p*= 0.0001) and IGHV1-2 (*p*= 0.0002) were significantly under-represented in Chinese CLL, whilst IGHV3-7 (*p*= 0.04) and IGHV4-39 (*p*= 0.004) were more recurrent among Chinese than Italian patients. In addition, the IGHV4-59 (*p*= 0.01), IGHV1-3 (*p*= 0.02) and IGHV3-74 (*p*= 0.04) genes were most typically used by Chinese CLL (12%) but not by the Italian cohort of CLL (5%) (*p*= 0.0001). IGHV3-21 showed a comparable frequency (3% Chinese vs 3.8% Italian CLL) (Figure 1B).

**Figure 2:**
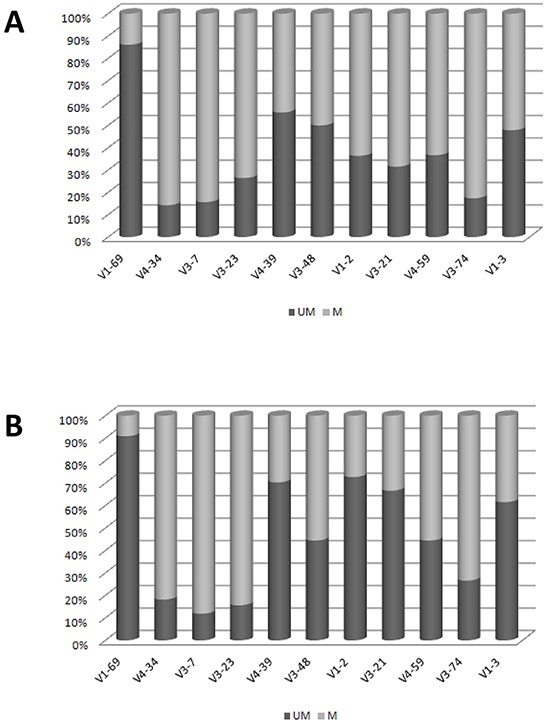
Mutational Patterns of IGHV genes **A.** Chinese CLL. **B.** Italian CLL. Data are represented as percentage.

The known pattern of mutations for each IGHV gene was largely conserved in both Chinese and Italian CLL. In fact, the IGHV1-69 gene carried few or no mutations, while IGHV4-34, IGHV3-7 and IGHV3-23 showed a high frequency of mutations in both cohorts. Contrariwise, IGHV3-21 (*p*= 0.002) and IGHV1-2 (*p*= 0.03) genes showed a higher frequency of mutations among Chinese than Italian CLL (Figure 2). In particular, IGHV3-21 showed a median of 9.4% (range 2.5-18.9%) mutations in Chinese CLL vs 4% (range 2.5-9%) in Italian CLL.

Also the comparison of IGHD subgroups and genes allowed to identify additional differences between the two CLL cohorts. The IGHD6 subgroup, the second most frequent after the IGHD3 family in Chinese CLL, was significantly increased compared to Italian CLL (*p*= 0.004). Similarly, there was a significant over-usage of IGHD3-10 (*p*= 0.002), IGHD6-13 (*p*= 0.0001) and IGHD2-21 (*p*= 0.03) genes and under-usage of IGHD3-3 (*p*= 0.0001) and IGHD2-2 (*p*= 0.02) genes in Chinese CLL.

As for the IGHJ subgroup, although the distribution was similar among the two populations, IGHJ6 (*p*= 0.002) was significantly under-represented in the Chinese series.

**Figure 3:**
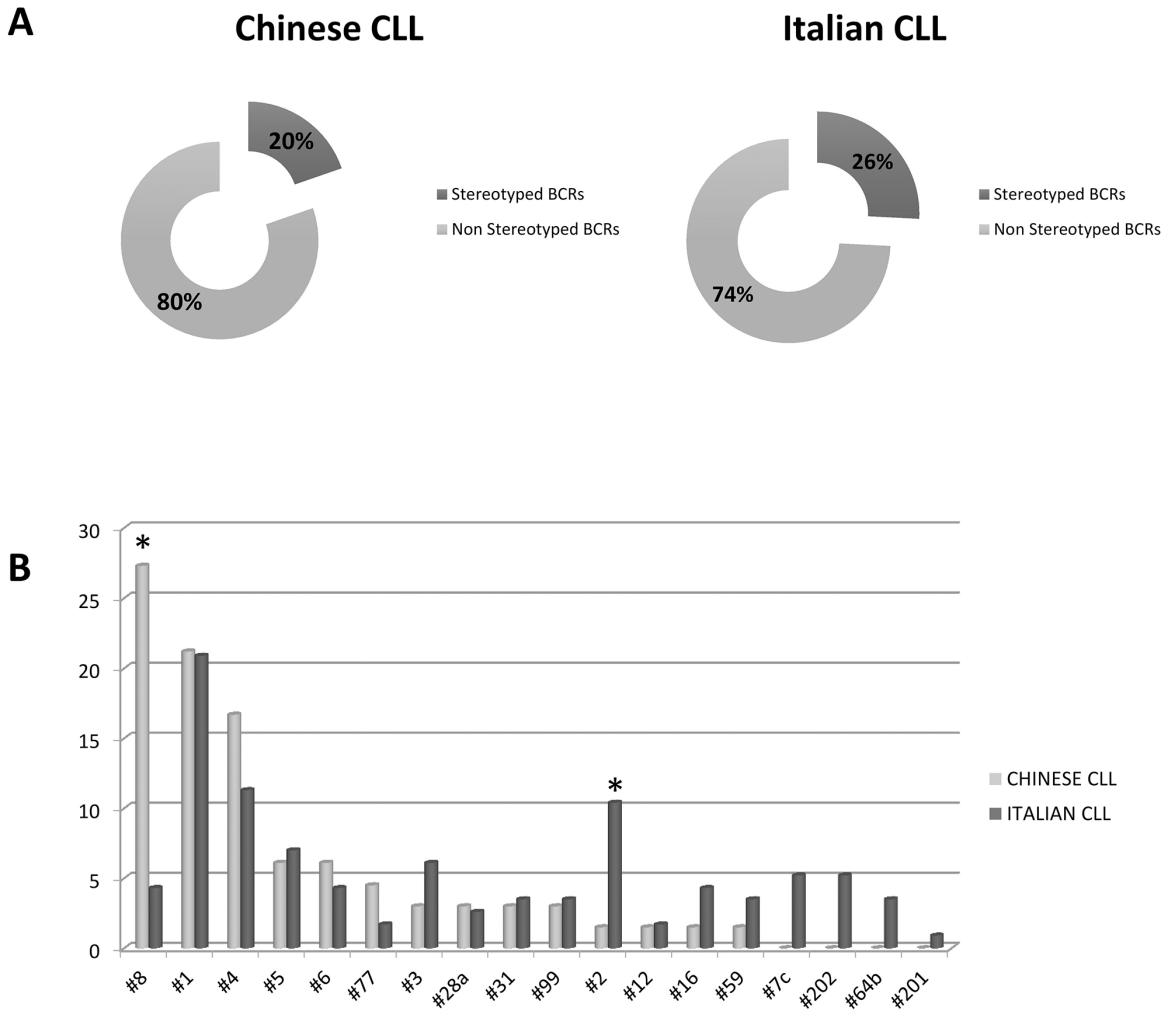
Stereotyped BCRs representation **A.** Chinese and Italian stereotyped and non-stereotyped BCRs. **B.** Frequency of Major subsets in Chinese and Italian series. * marks the significant differences (p values in the text). Data are represented as percentage.

**Figure 4:**
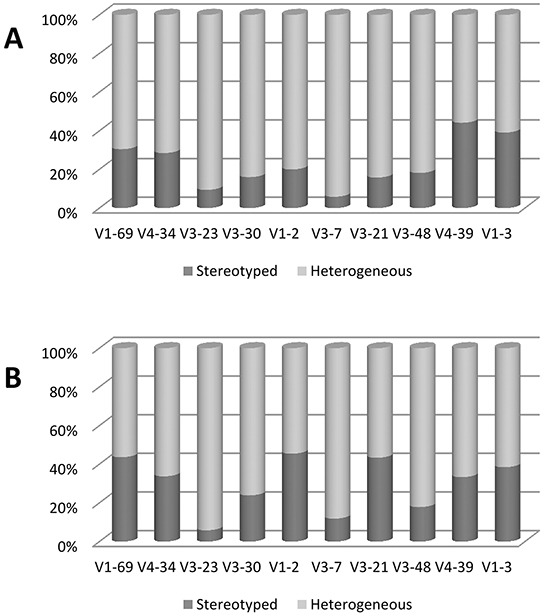
Frequency of stereotyped and heterogeneous BCRs for selected IGHV genes **A.** Chinese CLL. **B.** Italian CLL. Data are represented as percentage.

Comparative analysis of the HCDR3 sequences revealed notable differences. The proportion of known stereotyped receptors was significantly lower in Chinese (19.7%) than in Italian CLL (25.8%) (*p*= 0.007) (Figure 3A). In particular, within the major subsets, a significantly higher frequency of subset #8 (*p*= 0.0001) and lower frequency of subset #2 (*p*= 0.03) were observed in the Chinese cohort (Figure 3B). These features are due to the different frequencies of the stereotyped genes, given that most IGHV genes showed the same propensity to be used in stereotyped rearrangements in both series (Figure 4). In fact, the most represented stereotyped genes in Italian CLL were IGHV1-69> IGHV4-34> IGHV1-2> IGHV3-21> IGHV3-30> IGHV4-39, whilst being IGHV4-34> IGHV4-39> IGHV1-69> IGHV1-3> IGHV3-23> IGHV4-59 in Chinese CLL. In particular, the stereotyped HCDR3 sequences expressing IGHV1-69 and IGHV3-21 genes were significantly less represented in Chinese CLL than Italian CLL (*p*= 0.0006 and *p*= 0.0003, respectively); indeed, the IGHV3-21 gene exhibited a higher heterogeneity within its HCDR3 sequence in Chinese than Italian CLL (Figure 4).

### Clinical impact of IGHV features in Chinese CLL

Prognosis was evaluated on 612 Chinese CLL patients, with a median follow-up of 28 months (range: 0-270.9). The median overall survival (OS) was 121.7 months for 566 evaluable cases. Treatment-free interval (TFI) from diagnosis was 18.4 months (range: 0-194.8) for 580 evaluable cases, 327 of whom required treatment. Richter's transformation occurred in 16 of 575 evaluable cases (2.8%).

CLL in Binet stages A, B or C had a median OS of 165.1, 98 and 82.2 months, respectively (*p*= 0.0001).

Chinese CLL with unmutated IGHV showed a poor prognosis in terms of OS (85.1 vs 165.1 months, *p*<0.0001) and TFI (4.0 vs 46.1 months, *p*<0.0001) compared to mutated IGHV cases, as expected.

As reported for Caucasian CLL, subset #1 Chinese CLL (n=14) showed a poor prognosis in terms of OS (median OS 28.0 months vs 85.1 for unmutated IGHV vs 165.1 for mutated IGHV, *p*<0.0001) and TFI (median TFI 0.8 months vs 4.8 for unmutated IGHV vs 46.2 for mutated IGHV, *p*<0.0001), even worse than that of the other IGHV unmutated CLL.

Subset #8 CLL (n=18) showed an OS (median 82.2 months) and TFI (median 1.7 months) as poor as the other IGHV unmutated CLL, although there was a significant association with the occurrence of Richter's syndrome (n=3 out of 16) (*p*= 0.0095), as reported for Caucasian CLL.

The low number of events did not allow to analyze the prognostic impact of subset #4 (n=11).

Finally, the OS and TFI of Chinese patients with IGVH3-23 were not different from the other cases with mutated IGHV (*p*= 0.48 and *p*= 0.41, respectively). The low number of events did not allow to highlight a different outcome between stereotyped IGHV3-23 (n=7) and heterogeneous IGHV3-23 (n=32) cases.

## DISCUSSION

In Caucasian CLL, the extensive research on IGHVDJ rearrangements has shown that immunoglobulin gene usage is not random, but biased by the recurrence of certain IGHV genes as well as by the existence of subsets with stereotyped BCR [31, 32]. In contrast, little is known about the IGHV features of Asian CLL, although some differences have been reported in small cohorts of Chinese and Japanese patients [24, 25]. In order to define geographic and ethnic immunoglobulin gene variations, we characterized IGHVDJ rearrangements and HCDR3 stereotypy in a large series of Chinese CLL and compared them with Italian CLL. Our Italian cohort is representative of Caucasian CLL, being comparable with other Italian [30] and Western series [28].

A mutated IGHV status was more frequent in Chinese than in Italian CLL, thus consolidating previous observations made in small Asian series [24, 25]. This holds true also when considering patients in homogeneous phases of disease.

With regard to the IGHV subgroups, Chinese CLL showed an IGHV3>IGHV4>IGHV1 order of frequency, significantly different from Italian CLL patients, who showed an IGHV3>IGHV1>IGHV4 distribution. These results support those reported on both Chinese [24] and Japanese [25, 33] CLL, confirming that this family usage is typical of Asian CLL.

Focusing on the IGHV gene distribution, also Chinese CLL showed a non-random use of immunoglobulin genes, with the IGHV4-34 being the most represented. Moreover, IGHV1-69 and IGHV1-2 were significantly under-represented in Chinese CLL, whilst IGHV4-39 and IGHV3-7 were more recurrent in Chinese than Italian CLL. Furthermore, the IGHV4-59, IGHV3-74 and IGHV1-3 genes were common in Chinese CLL, whilst rare in Italian CLL.

The known pattern of mutations for each specific IGHV gene was largely conserved, i.e. the IGHV1-69 gene shows analogous molecular features in Caucasian and in Chinese CLL, being unmutated and representing the most frequent gene of the IGHV1 subgroup [28, 34]. Thus, the low recurrence of the IGHV1-69 gene in Chinese CLL largely accounts for the lower overall frequency of the IGHV1 subgroup and of unmutated cases. On the other hand, the high incidence of the IGHV3-7, IGHV4-39 and IGHV3-74 genes could be responsible of the high representation of mutated IGHV, since these genes often show a mutated profile. Relevant exceptions are represented by the IGHV1-2 gene, that was significantly more mutated in Chinese than in Italian CLL, and the IGHV3-21 gene. The latter, equally represented in Chinese (3%) and Italian CLL (3.8%), was more frequently mutated in China.

Our results reinforce those reported in other small Asian CLL series [24, 25], especially with regard to IGHV1-69 and IGHV3-7, strongly supporting the contribution of ethnic and geographic parameters in shaping the IGHV repertoire. Moreover, the same pattern is also reported in Iranian CLL [27], suggesting that this geographic-related variation is already established in the Middle-East. Thus, this different pattern between Eastern and Western CLL adds to the other known geographic variation related to the IGHV3-21 gene, over-represented in Northern European CLL compared to Mediterranean CLL [29].

We also identified additional differences in the frequencies of IGHD and IGHJ genes. Among others, a significant under-usage of IGHD3-3 and IGHJ6 genes was observed in the Chinese cohort. Given the specific combination of IGHV1-69 with the IGHD3-3 and IGHJ6 genes in Caucasian CLL [28, 34], their low occurrence in Chinese CLL could be related to the low frequency of IGHV1-69. To our knowledge, these results have never been previously reported.

The analysis of HCDR3 sequences proved the presence of stereotyped BCR in one-third of Caucasian CLL [28, 30, 31], suggesting the recognition of a common antigenic determinant [35]. We therefore characterized the HCDR3 sequences in Chinese CLL, which have not been investigated so far, for their potentially relevant pathogenetic implications. Chinese CLL showed a significantly lower representation of stereotyped sequences (19.7%) compared to Italian CLL (25.8%). The stereotyped subsets defined major among Caucasian CLL collectively were over-represented also in the Chinese cohort, but with a significantly higher frequency of subset #8 (China vs Italy: 27.3% vs 4.3%) and a lower frequency of subset #2 (China vs Italy: 1.5% vs 10.4%). Subset #8, the most represented Chinese stereotype, is characterized by the use of the IGHV4-39 gene, which is indeed one of the most recurrent gene in Chinese CLL. On the contrary, the low frequency of subset #2 is due to the heterogeneous HCDR3 of IGHV3-21 in Chinese CLL, whilst in Caucasian CLL the IGHV3-21 gene is mostly stereotyped [29]. The low frequency of subset #2 in Chinese CLL could parallel the recently reported low incidence of *SF3B1* mutations [23], in line with their reported association in Caucasian CLL [36]. Of note, the clinical impact of CLL stereotypy is independent of the ethnic context, since in Chinese CLL subset #1 displayed a poor prognosis and subset #8 was related to an increased risk of transformation into Richter syndrome, similarly to Caucasian CLL [37, 38].

With the exception of the IGHV3-21 gene, it is worth noting that all the other IGHV genes (i.e. IGHV1-69, IGHV4-39, etc) maintain the same proportion of homologous/heterologous BCR both in Chinese and in Italian CLL. Thus, the determinant of the lower frequency of stereotyped BCR in Chinese CLL could be due to the prevalence of certain genes (i.e. IGHV3-7 and IGHV3-74) and the reduced frequency of others (i.e. IGHV1-69), each maintaining the same tendency to stereotypy.

By clustering analysis, we detected 16 new paired clusters among Chinese cases, characterized by the remarkable recurrence of the IGHV3-23 gene; contrariwise, in Caucasian CLL this gene constantly exhibits an heterogeneous HCDR3 [39]. Moreover, IGHV3-23 gene usage is considered an independent negative prognosticator within Caucasian mutated CLL [39], but this was not the case among Chinese CLL.

In line with the proposal of Ghia et al. for Caucasian CLL [29], also in Chinese CLL the IGHV pattern may be due to either a specific genetic background and/or to the effects of potential environmental variables. The observation of new paired clusters in Chinese CLL, never reported in Caucasian CLL, supports the hypothesis of an Asian-specific antigenic selection at least in some cases. To further investigate the importance of the genetic background in determining the occurrence of different rearrangements in distinct geographic and/or ethnic groups, the normal IGHV repertoire of healthy subjects of the same ethnicity and from the same geographic areas should also be investigated. Although some evidences are reported for Italians [40], at present no data on the normal repertoire are available for Chinese population.

In conclusion, the results hereby described offer the most extensive catalogue of the BCR features of Chinese CLL, being based on the largest series of Chinese CLL so far reported. In comparison to Italian CLL, most of the Chinese IGHV genes show different frequencies, but maintain the same propensity towards mutations and stereotypy, with the same clinical impact. The distinctive molecular features of IGHV3-21 and IGHV3-23 Chinese CLL genes deserve further investigation.

## MATERIALS AND METHODS

### Patients

A series of 623 patients diagnosed with CLL from 3 different institutions situated in China - Nanjing (338 cases) and Tianjin (166 cases) - and Hong Kong (119 cases), were included in the study. A comparison of the three CLL groups, all of Han Chinese ethnicity, showed a high degree of internal similarities with regard to the somatic hypermutation status and IGHVDJ rearrangements. Therefore, the whole cohort of Chinese CLL was compared to an Italian cohort of 789 CLL patients collected between 2001 and 2014 at the Hematology Institute of the “Sapienza” University of Rome. The Italian CLL cohort was comparable to the Mediterranean series [29,30], and to those reported by Agathangelidis et al [28] (Supplementary Table S1).

All satisfied the morphologic and immunophenotypic diagnostic criteria for CLL [41]. The characteristics of the Chinese and Italian patients are summarized in Table 1. Chinese CLL appear older than Italians (*p*<0.001) and in more advanced stages (more stage C) (*p*= 0.0194). Regarding FISH lesions, they show less 11q- (*p*= 0.0082) and 13q-only (*p*= 0.0026), more trisomy12 (*p*= 0.0273) and more 17p- (*p*= 0.0457) than Italian CLL. This is in contrast with the disease phase at study, which included more pre-treated patients in the Italian series. After excluding pre-treated patients from both cohorts, we confirm all the above mentioned differences (age: *p*= 0.0001; stage C: *p*= 0.0001; 11q-: *p*= 0.012; 13q only: *p*= 0.028; 17p-: *p*= 0.0108), with the exception of trisomy 12 (*p*= 0.3). No difference was observed in the WBC count: 27.5 × 10^9/L (range 2.1-452.8 × 10^9/L) in Chinese CLL and 30.3 × 10^9/L (range 1.7-622.9 × 10^9/L) in Italian CLL (*p*= 0.52).

**Table 1:** Clinical and biological features of Chinese and Italian CLL patients (* cases with available clinical information)

	Chinese CLL (N=611*)	Italian CLL (N=789)	Chinese CLL untreated (N=536)	Italian CLL untreated (N=595)
**Sex, M/F**	412/190*	520/258*	362/171*	387/199*
** Median age, y (range) at diagnosis**	**60 (16-94)**	**55 (26-87)**	**60 (16-94)**	**55 (26-87)**
**Stage at study**	**N=575**	**N=546**	**N=517**	**N=461**
** A**	245 (42.6%)	271 (49.6%)	232	251
** B**	141 (24.5%)	181 (33.2%)	127	144
** C**	187 (32.5%)	94 (17.2%)	156	66
** Richter**	2 (0.4%)	-	2	-
**Disease phase at study**	**N=594**	**N=729**	**N=536**	**N=595**
** Diagnosis**	290 (48.8%)	304 (41.7%)	290	304
** First progression**	246 (41.4%)	291 (39.9%)	246	291
** Subsequent progression (post-treatment)**	58 (9.8%)	134 (18.4%)	-	-
**FISH**	**N=373**	**N=628**	**N=329**	**N=535**
** del17p**	45 (12.1%)	51 (8.12%)	38	34
** del11q**	38 (10.2%)	102 (16.24%)	31	82
** Tris12**	68 (18.2%)	81 (12.9%)	50	68
** del13q only**	98 (26.3%)	223 (35.51%)	101	204
** Normal FISH**	124 (33.2%)	171 (27.23%)	109	147
**IGHV**	**N=611**	**N=789**	**N=536**	**N=595**
** Germline**	**214**	402	167	283
** Mutated**	**397**	387	369	312

### Analysis of immunoglobulin rearrangements and sequence analysis

In all cases, the analysis of IGHVDJ genes was carried out on leukemic cells obtained from peripheral blood samples after isolation by Ficoll gradient or buffy-coat. PCR amplification and sequence analysis of IGHVDJ rearrangements were performed on either genomic DNA (gDNA) or cDNA using sense family-specific VH primers (framework region 1 [FR1] or VH leader primers), combined with consensus JH primers as previously described [42] or following the IGH Somatic Hypermutation Assay v2.0 protocol (InVivoScribe) [24]. PCR products were sequenced directly or after a cloning procedure, using 3130 Genetic Analyzer (Life Technologies, Carlsbad, CA).

Productive rearrangements were analyzed by IMGT database (http://imgt.cines.fr, Montepellier, France) [43] and the IMGT/V-QUEST tool (version 3.3.0) [44].

The following features were evaluated for all IGHVDJ rearrangements: IGHV gene and allele usage, percentage of identity to the closest germline IGHV allele, HCDR3 length and composition calculated between codons 107 and 117, and IGHD-IGHJ gene usage.

To identify clusters of sequences with common HCDR3 motifs, we evaluated the HCDR3 region by the multiple sequence alignment ClustalW2 software (http://www.ebi.ac.uk), followed by a manual curation. Clustering was performed comparing our HCDR3 sequences to those present in literature's databases [28, 45, 36].

To identify subsets of similar HCDR3, we used several criteria. First, for major subsets we followed the criteria proposed by Agathangelidis et al. [28] : *i)* sharing at least 50% amino acid identity and 70% similarity calculated through common sequence patterns; *ii)* having identical HCDR3 lengths and identical offsets of shared patterns between sequences; and *iii)* carrying IGHV genes of the same clan. Second, we identified homologous HCDR3 as those which shared a HCDR3 homology equal to or exceeding 60%, regardless of the usage of the IGHV gene: groups of 2 CLL cases with homologous HCDR3 were defined as paired clusters; clusters of 3 or more cases were defined as subsets. Known stereotyped HCDR3s were defined and named according to published criteria: for major subsets by Agathangelidis et al. [28]; for minor subsets by Murray et al. [45] and for novel subsets by Rossi et al. [36].

### Statistical analysis

Characteristics of patients were summarized by means of cross-tabulations (categorical variables), quantiles (median etc; for ordinal factors) or by means of standard positional and variation parameters (mean, standard deviation; for continuous variables). Non-parametric tests were applied, in univariate analysis, for comparisons between groups (Chi-Squared and Fisher Exact test for difference in terms of categorical variables or response rate, Mann-Whitney and Kruskal-Wallis test for difference in terms of continuous variables).

Overall survival was estimated using the Kaplan-Meier Product Limit estimator. Differences were evaluated by means of Log-Rank test after assessment of proportionality of hazards.

TFI was estimated using the proper non-parametric method; the Gray test was applied for significance tests on cumulative incidence curves.

All the analyses were performed using the SAS system software (version 9.4); all tests were two-sided, at a significance level of 0.05 and confidence intervals were calculated at 95% level.

## SUPPLEMENTARY TABLES






