# Supplementary material for: Immunoglobulin gene rearrangements in Chinese and Italian patients with chronic lymphocytic leukemia
Source: Oncotarget. 2016 Mar 1;7(15):20520–31. doi: 10.18632/oncotarget.7819 (PMC4991472; doi:10.18632/oncotarget.7819)
Supplement: Supplementary file 1 [file oncotarget-07-20520-s001.pdf]

SUPPLEMENTARY TABLES

Supplementary Table S1: Italian CLL IGHV repertoire - IGHV repertoire of Italian CLL (IGHV genes, mutations and stereotypy)

See Supplementary File 1

Supplementary Table S2: Chinese CLL IGHV repertoire - IGHV repertoire of Chinese CLL (IGHV genes, mutations and stereotypy)

See Supplementary File 2
