# Supplementary material for: Immunoglobulin gene rearrangements in Chinese and Italian patients with chronic lymphocytic leukemia
Source: Oncotarget. 2016 Mar 1;7(15):20520–31. doi: 10.18632/oncotarget.7819 (PMC4991472; doi:10.18632/oncotarget.7819)
Supplement: Supplementary file 2 [file oncotarget-07-20520-s002.doc]

Supplementary Table S1A: Italian IGHV Mutational status

| Mutational status | Italian No | % |
| --- | --- | --- |
| M | 387 | 48,9 |
| UM | 405 | 51,1 |
| totals | 792 | 100,0 |


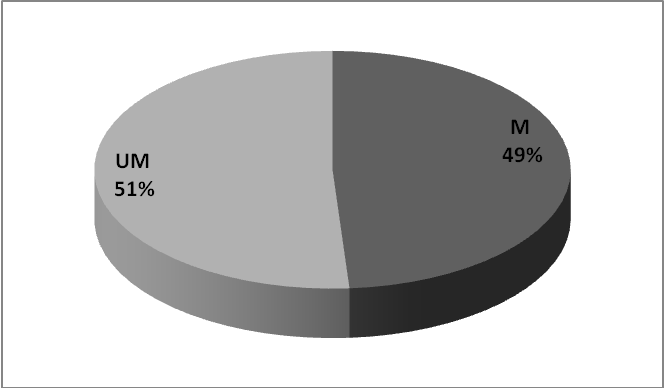


Supplementary Table S1B: Italian IGHV subgroup and gene repertoire

| IGHV Subgroup | Italian No | % |
| --- | --- | --- |
| IGHV1 | 199 | 25,1 |
| IGHV2 | 26 | 3,3 |
| IGHV3 | 364 | 46,0 |
| IGHV4 | 163 | 20,6 |
| IGHV5 | 25 | 3,2 |
| IGHV6 | 9 | 1,1 |
| IGHV7 | 6 | 0,8 |
| totals | 792 | 100,0 |


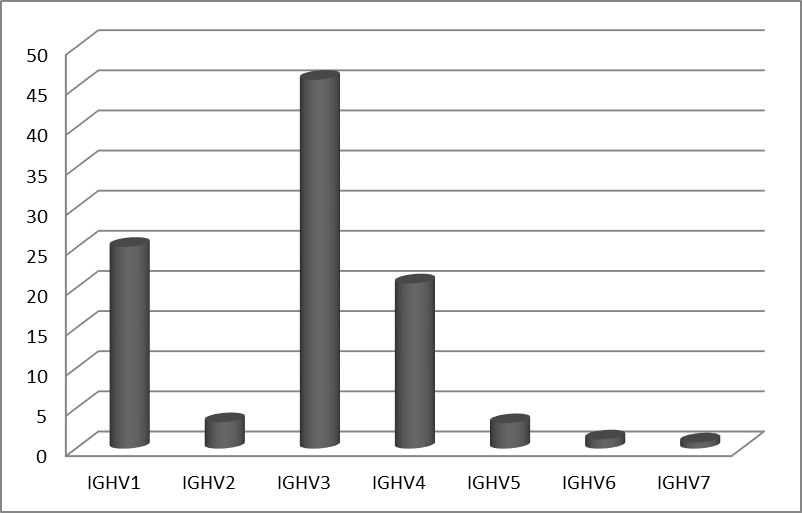


| IGHV gene | Italian No | % |
| --- | --- | --- |
| V1-18 | 10 | 1,3 |
| V1-2 | 44 | 5,6 |
| V1-24 | 2 | 0,3 |
| V1-3 | 13 | 1,6 |
| V1-46 | 10 | 1,3 |
| V1-58 | 1 | 0,1 |
| V1-69 | 110 | 13,9 |
| V1-8 | 9 | 1,1 |
| V2-05 | 22 | 2,8 |
| V2-26 | 3 | 0,4 |
| V2-70 | 1 | 0,1 |
| V3-11 | 31 | 3,9 |
| V3-13 | 1 | 0,1 |
| V3-15 | 15 | 1,9 |
| V3-20 | 4 | 0,5 |
| V3-21 | 30 | 3,8 |
| V3-23 | 51 | 6,4 |
| V3-30 | 46 | 5,8 |
| V3-30-3 | 9 | 1,1 |
| V3-33 | 27 | 3,4 |
| V3-35 | 1 | 0,1 |
| V3-43 | 1 | 0,1 |
| V3-48 | 28 | 3,5 |
| V3-49 | 12 | 1,5 |
| V3-53 | 19 | 2,4 |
| V3-64 | 5 | 0,6 |
| V3-66 | 3 | 0,4 |
| V3-7 | 42 | 5,3 |
| V3-72 | 7 | 0,9 |
| V3-73 | 6 | 0,8 |
| V3-74 | 15 | 1,9 |
| V3-9 | 11 | 1,4 |
| V4-30-2 | 3 | 0,4 |
| V4-30-4 | 4 | 0,5 |
| V4-31 | 11 | 1,4 |
| V4-34 | 77 | 9,7 |
| V4-38-2 | 0 | 0 |
| V4-39 | 27 | 3,4 |
| V4-4 | 12 | 1,5 |
| V4-59 | 18 | 2,3 |
| V4-61 | 11 | 1,4 |
| V5-10-1 | 5 | 0,6 |
| V5-51 | 20 | 2,5 |
| V6-1 | 9 | 1,1 |
| V7-4-1 | 5 | 0,6 |
| V7-81 | 1 | 0,1 |
| totals | 792 | 100 |


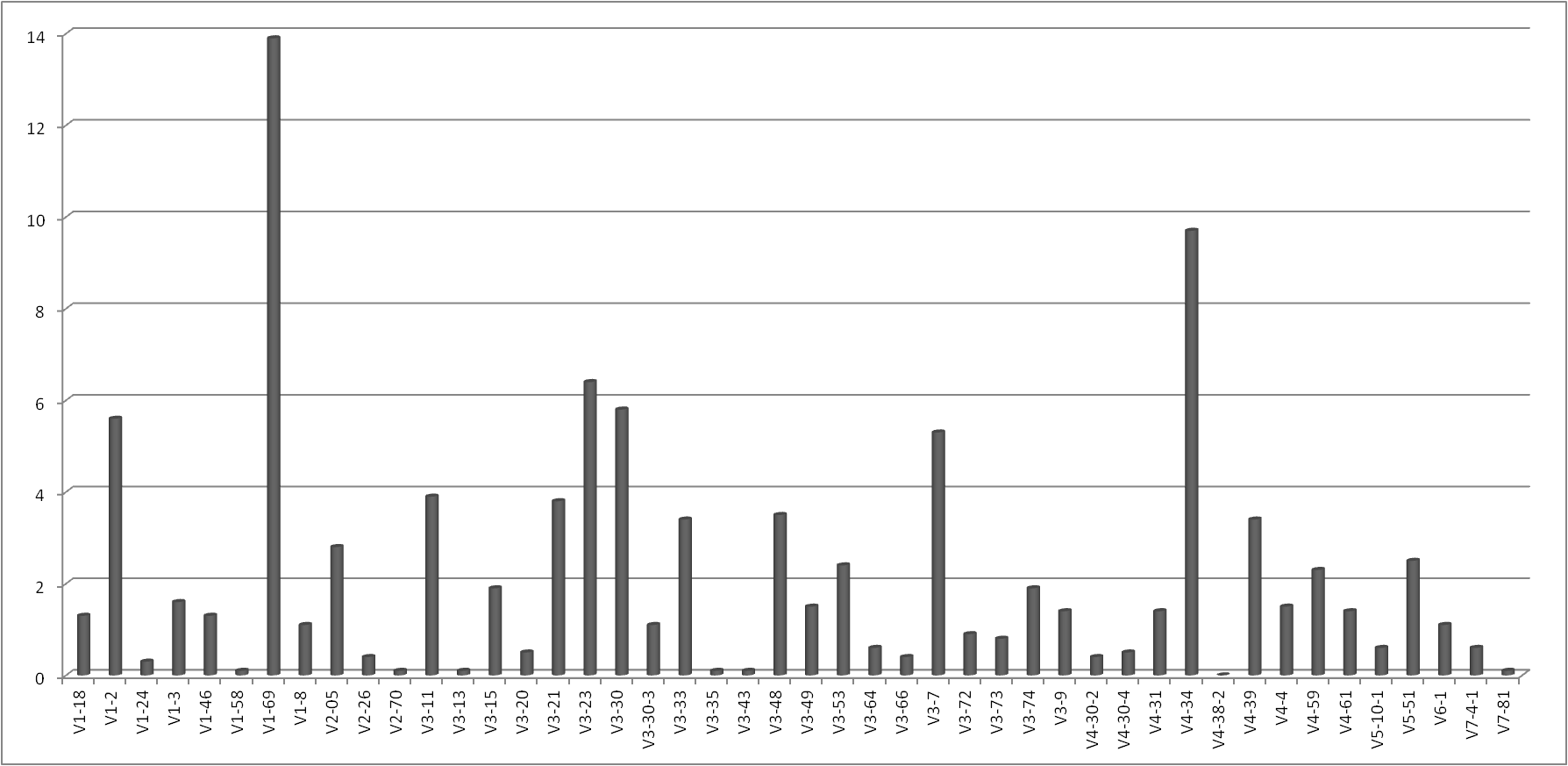


Supplementary Table S1C: Italian IGHD subgroup and gene repertoire

| IGHD Subgroup | Italian No | % |
| --- | --- | --- |
| IGHD1 | 65 | 8,4 |
| IGHD2 | 135 | 17,4 |
| IGHD3 | 337 | 43,5 |
| IGHD4 | 60 | 7,8 |
| IGHD5 | 62 | 8 |
| IGHD6 | 107 | 13,8 |
| IGHD7 | 8 | 1 |
| totals | 774 | 100 |


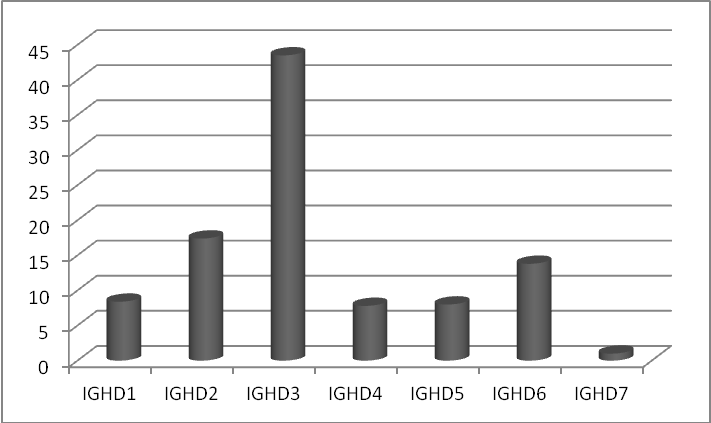


| IGHD gene | Italian No | % |
| --- | --- | --- |
| IGHD1-1 | 8 | 1 |
| IGHD1-14 | 6 | 0,8 |
| IGHD1-20 | 8 | 1 |
| IGHD1-26 | 35 | 4,5 |
| IGHD1-7 | 8 | 1 |
| IGHD2-15 | 36 | 4,7 |
| IGHD2-2 | 74 | 9,6 |
| IGHD2-21 | 18 | 2,3 |
| IGHD2-8 | 8 | 1 |
| IGHD3-10 | 55 | 7,1 |
| IGHD3-16 | 36 | 4,7 |
| IGHD3-22 | 82 | 10,6 |
| IGHD3-3 | 133 | 17,2 |
| IGHD3-9 | 31 | 4 |
| IGHD4-11 | 10 | 1,3 |
| IGHD4-17 | 31 | 4 |
| IGHD4-23 | 16 | 2,1 |
| IGHD4-4 | 3 | 0,4 |
| IGHD5-12 | 25 | 3,2 |
| IGHD5-18 | 14 | 1,8 |
| IGHD5-24 | 18 | 2,3 |
| IGHD5-5 | 5 | 0,6 |
| IGHD6-13 | 28 | 3,6 |
| IGHD6-19 | 68 | 8,8 |
| IGHD6-25 | 3 | 0,4 |
| IGHD6-6 | 7 | 0,9 |
| IGHD7-27 | 8 | 1 |
| totals | 774 | 100 |


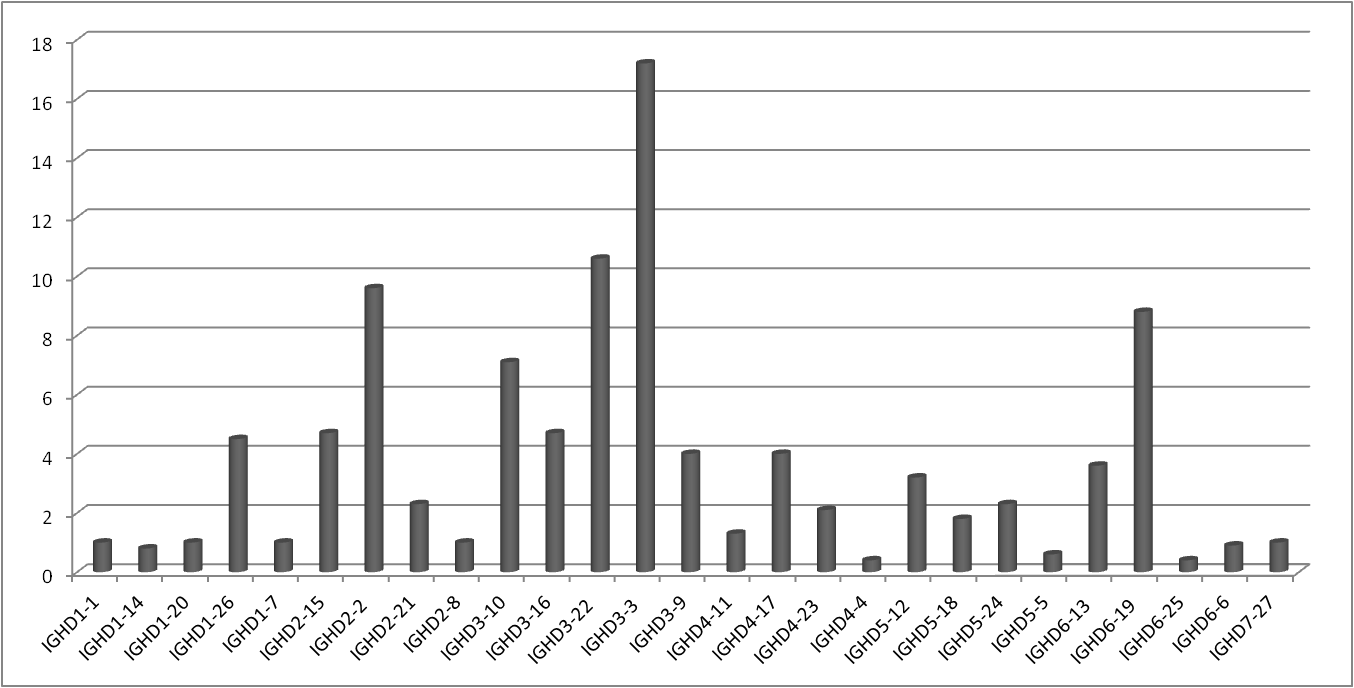


Supplementary Table S1D: Italian IGHJ gene repertoire

| IGHJ gene | Italian No | % |
| --- | --- | --- |
| IGHJ1 | 14 | 1,8 |
| IGHJ2 | 15 | 1,9 |
| IGHJ3 | 78 | 9,8 |
| IGHJ4 | 332 | 41,9 |
| IGHJ5 | 89 | 11,2 |
| IGHJ6 | 264 | 33,3 |
| totals | 792 | 100 |


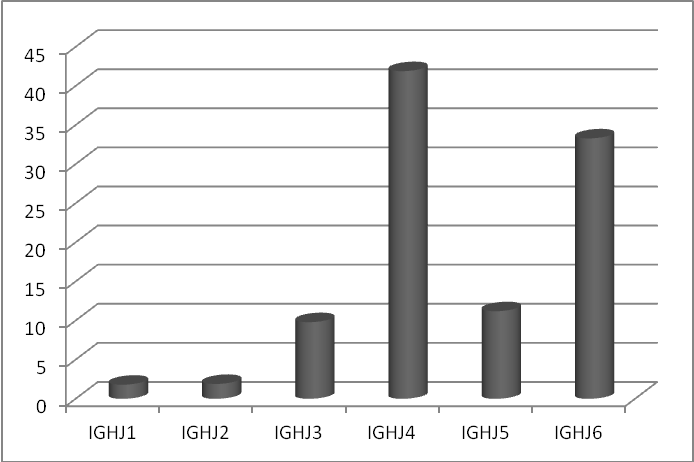


Supplementary Table S1E: Italian Stereotyped and Non-Stereotyped BCRs and Major Subsets

| Italian CLL | No | % |
| --- | --- | --- |
| Stereotyped BCRs | 204 | 25,8 |
| Non Stereotyped BCRs | 588 | 74,2 |
| totals | 792 | 100 |


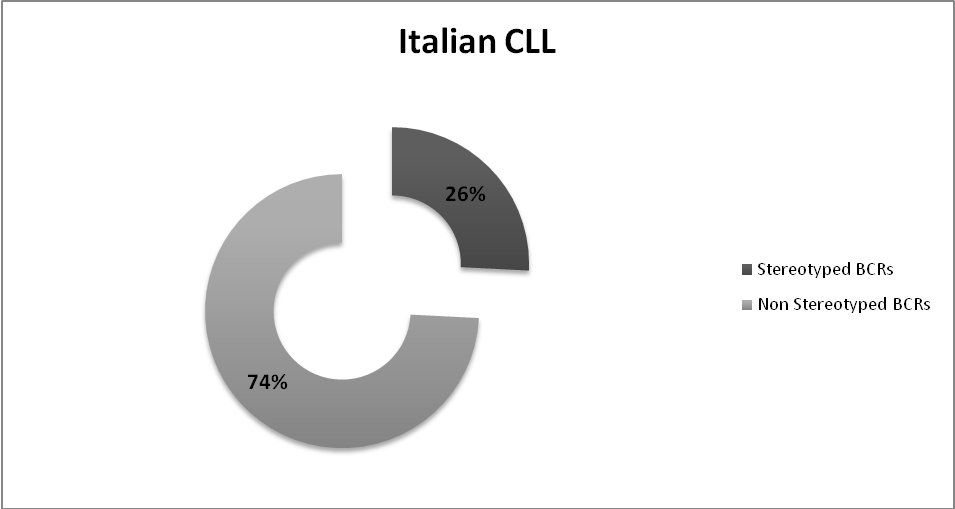


| IGHV gene(s) | Major Subset | No | % |
| --- | --- | --- | --- |
| #1 | Clan I V genes | 24 | 20,9 |
| #4 | V4-34 | 13 | 11,3 |
| #2 | V3-21 | 12 | 10,4 |
| #5 | V1-69 | 8 | 7 |
| #3 | V1-69 | 7 | 6,1 |
| #7c | V1-69 | 6 | 5,2 |
| #202 | Clan III V genes | 6 | 5,2 |
| #6 | V1-69 | 5 | 4,3 |
| #8 | V4-39 | 5 | 4,3 |
| #16 | V4-34 | 5 | 4,3 |
| #31 | Clan III V genes | 4 | 3,5 |
| #59 | Clan I V genes | 4 | 3,5 |
| #64b | V3 (V3-48) | 4 | 3,5 |
| #99 | Clan I V genes | 4 | 3,5 |
| #28a | V1 (V1-2) | 3 | 2,6 |
| #12 | V1-2, V1-46 | 2 | 1,7 |
| #77 | V4 (V4-4, V4-59) | 2 | 1,7 |
| #201 | V4-34 | 1 | 0,9 |
| #14 | V4 (V4-4) | 0 | 0 |
| totals |  | 115 | 100 |


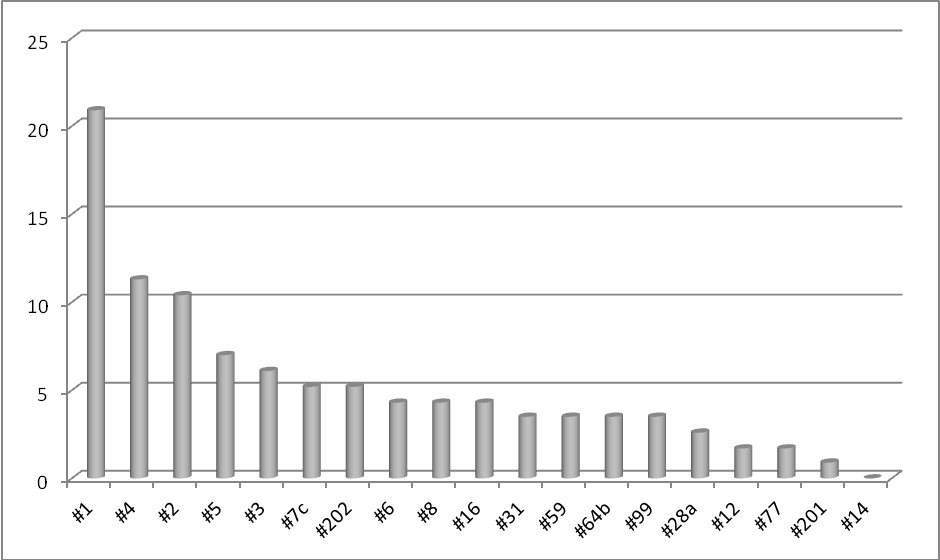


Supplementary Table S1F: Characteristics of the Italian subsets

| **Sample** | **Subset No** | **IGHV gene(s)** | **IGHD gene(s)** | **IGHJ gene(s)** | | **Mutational status** | **VH CDR3 length** | **HCDR3 AMINOACIDS** |
| --- | --- | --- | --- | --- | --- | --- | --- | --- |
| ROME_001 | #1 | IGHV1-2 | IGHJ4 | | IGHD6-19 | unmutated | 15 | CARAQWLVMVSFDYW |
| ROME_048 | #1 | IGHV1-2 | IGHJ4 | | IGHD6-19 | unmutated | 15 | CARVQWLGPNSFDYW |
| ROME_062 | #1 | IGHV1-3 | IGHJ4 | | IGHD6-19 | unmutated | 15 | CAREQWLVLPYFDYW |
| ROME_063 | #1 | IGHV3-49 | IGHJ4 | | IGHD3-22 | mutated | 15 | CAREQWLVLPYFDYW |
| ROME_080 | #1 | IGHV1-8 | IGHJ4 | | IGHD3-10 | unmutated | 16 | CARGQWFGEYYFDYW |
| ROME_161 | #1 | IGHV1-2 | IGHJ4 | | *no D identified* | unmutated | 16 | CARDQWVGMAEPFDYW |
| ROME_258 | #1 | IGHV5-51 | IGHJ4 | | IGHD6-19 | unmutated | 15 | CARLQWLPQDYFDYW |
| ROME_288 | #1 | IGHV1-2 | IGHJ4 | | IGHD6-19 | unmutated | 15 | CAREQWLVLLHFDYW |
| ROME_319 | #1 | IGHV1-2 | IGHJ4 | | IGHD6-19 | unmutated | 15 | CAREQWLVLNHFDYW |
| ROME_403 | #1 | IGHV5-10-1 | IGHJ4 | | IGHD6-19 | unmutated | 15 | CAREQWLTRINYDYW |
| ROME_416 | #1 | IGHV1-18 | IGHJ4 | | IGHD5-12 | unmutated | 15 | CARAQWLPIYYFDYW |
| ROME_538 | #1 | IGHV1-3 | IGHJ4 | | IGHD6-19 | unmutated | 15 | CAREQWLVLNYFDYW |
| ROME_543 | #1 | IGHV1-18 | IGHJ4 | | IGHD2-15 | unmutated | 15 | CARIQWWLVRYFDYW |
| ROME_552 | #1 | IGHV1-3 | IGHJ4 | | IGHD6-19 | unmutated | 15 | CARKQWLTTGYFDYW |
| ROME_556 | #1 | IGHV1-2 | IGHJ4 | | IGHD6-19 | unmutated | 15 | CARRQWLVLDNFDYW |
| ROME_571 | #1 | IGHV1-8 | IGHJ4 | | IGHD6-19 | unmutated | 15 | CARGQWLVLYNLDYW |
| ROME_577 | #1 | IGHV1-2 | IGHJ4 | | IGHD6-19 | unmutated | 15 | CAREQWLVLKNFDYW |
| ROME_586 | #1 | IGHV1-8 | IGHJ4 | | IGHD6-19 | unmutated | 15 | CARVQWLVRNNFDYW |
| ROME_608 | #1 | IGHV7-4-01 | IGHJ4 | | IGHD6-19 | unmutated | 15 | CAREQWLVVAHFDYW |
| ROME_620 | #1 | IGHV1-3 | IGHJ4 | | IGHD5-12 | unmutated | 15 | CARDQWLPTTYFDYW |
| ROME_677 | #1 | IGHV1-2 | IGHJ4 | | IGHD6-19 | unmutated | 15 | CARAQWLVIPYFDYW |
| ROME_713 | #1 | IGHV1-2 | IGHJ4 | | IGHD6-19 | unmutated | 15 | CAREQWLVLNHFDYW |
| ROME_746 | #1 | IGHV1-2 | IGHJ4 | | IGHD6-19 | unmutated | 15 | CAREQWLVLNHFDYW |
| ROME_759 | #1 | IGHV1-8 | IGHJ4 | | IGHD6-19 | unmutated | 15 | CARGQWWLVNYFDYW |
| ROME_128 | #2 | IGHV3-21 | IGHJ6 | | IGHD3-10 | mutated | 11 | CARDVNTMDVW |
| ROME_175 | #2 | IGHV3-21 | IGHJ6 | | IGHD1-1 | mutated | 11 | CARDKSDMDVW |
| ROME_212 | #2 | IGHV3-21 | IGHJ6 | | IGHD5-24 | unmutated | 11 | CATDRNAMDVW |
| ROME_351 | #2 | IGHV3-21 | IGHJ6 | | *no D identified* | mutated | 11 | CARDQTAMDVW |
| ROME_459 | #2 | IGHV3-30 | IGHJ6 | | IGHD5-24 | mutated | 11 | CARDAYGMDVW |
| ROME_483 | #2 | IGHV3-21 | IGHJ6 | | IGHD1-14 | mutated | 11 | CVTDRNGMDVW |
| ROME_498 | #2 | IGHV3-21 | IGHJ5 | | IGHD4-17 | unmutated | 11 | CARDETAYGDW |
| ROME_535 | #2 | IGHV3-21 | IGHJ6 | | IGHD1-14 | unmutated | 11 | CATDRNAMDVW |
| ROME_604 | #2 | IGHV3-21 | IGHJ6 | | IGHD5-24 | unmutated | 11 | CARDQDAMDVW |
| ROME_644 | #2 | IGHV3-21 | IGHJ6 | | IGHD5-24 | unmutated | 11 | CARDANAMDVW |
| ROME_770 | #2 | IGHV3-21 | IGHJ6 | | *no D identified* | unmutated | 11 | CATDRNGMDVW |
| ROME_788 | #2 | IGHV3-21 | IGHJ6 | | IGHD2-15 | unmutated | 11 | CARDANAMDVW |
| ROME_018 | #3 | IGHV1-69 | IGHJ6 | | IGHD2-2 | unmutated | 24 | CARGGDIVVVPAAMSYYYYGMDVW |
| ROME_066 | #3 | IGHV1-69 | IGHJ6 | | IGHD2-2 | unmutated | 23 | CARVVPDIVVVPAVYYYYGMDVW |
| ROME_140 | #3 | IGHV1-69 | IGHJ6 | | IGHD2-2 | unmutated | 23 | CARVVPDIVVVPAFYYYYGMDVW |
| ROME_610 | #3 | IGHV1-69 | IGHJ6 | | IGHD2-2 | unmutated | 24 | CARDRPDIVVVPAAISRYYGMDVW |
| ROME_692 | #3 | IGHV1-69 | IGHJ6 | | IGHD2-2 | unmutated | 24 | CAREIPDIVVVPADVYYYYGMDVW |
| ROME_740 | #3 | IGHV1-69 | IGHJ6 | | IGHD2-2 | unmutated | 24 | CARDLPDIVVVPAAIFRYYGMDVW |
| ROME_751 | #3 | IGHV1-69 | IGHJ6 | | IGHD2-2 | unmutated | 24 | CARSRPDIVVVPADIYYYYGMDVW |
| ROME_033 | #4 | IGHV4-34 | IGHJ6 | | IGHD2-2 | mutated | 22 | CARGYPDTAVVKRYYFYGMDVW |
| ROME_088 | #4 | IGHV4-34 | IGHJ6 | | IGHD4-17 | mutated | 22 | CARGYGDTPELKRYYYYGLDVW |
| ROME_103 | #4 | IGHV4-34 | IGHJ6 | | IGHD5-18 | mutated | 22 | CARGYPDTAVVKRYYYYGMDAW |
| ROME_172 | #4 | IGHV4-34 | IGHJ6 | | IGHD5-18 | mutated | 22 | CARGFGDTAVVRRYYYYGMDVW |
| ROME_179 | #4 | IGHV4-34 | IGHJ6 | | IGHD4-17 | mutated | 22 | CVRGYGDSPSLKRYYYYGLDVW |
| ROME_263 | #4 | IGHV4-34 | IGHJ6 | | IGHD3-22 | mutated | 22 | CARGYGDTVESRRYYYYGLDVW |
| ROME_422 | #4 | IGHV4-34 | IGHJ6 | | IGHD5-12 | mutated | 22 | CARGYGNTPDVRRYYYYGMDVW |
| ROME_431 | #4 | IGHV4-34 | IGHJ6 | | IGHD5-5 | mutated | 22 | CARGYPDTAVIRRYYYYGLDVW |
| ROME_509 | #4 | IGHV4-34 | IGHJ6 | | IGHD3-22 | mutated | 22 | CARGYGTDATTRRYYYYGMDVW |
| ROME_515 | #4 | IGHV4-34 | IGHJ6 | | IGHD3-22 | mutated | 22 | CARGWGDTAVVKRYYYYGMDVW |
| ROME_523 | #4 | IGHV4-34 | IGHJ6 | | *no D identified* | mutated | 22 | CARGWGTSPTHRRYYYYGMDVW |
| ROME_578 | #4 | IGHV4-34 | IGHJ6 | | IGHD4-23 | mutated | 22 | CATGYGVDPTVRRYYYYGMDVW |
| ROME_697 | #4 | IGHV4-34 | IGHJ6 | | IGHD5-18 | mutated | 22 | CARGWGDTAVVRRYYYYGMDVW |
| ROME_010 | #5 | IGHV1-69 | IGHJ6 | | IGHD3-10 | unmutated | 22 | CARAMVRGVINIYYYYYYMDVW |
| ROME_053 | #5 | IGHV1-69 | IGHJ6 | | IGHD3-10 | unmutated | 23 | CARTMVRGVILADYYYYYYMDVW |
| ROME_061 | #5 | IGHV1-69 | IGHJ6 | | IGHD3-10 | unmutated | 22 | CARVEVRGVIIFSLSYYGMDVW |
| ROME_163 | #5 | IGHV1-69 | IGHJ6 | | IGHD5-12 | unmutated | 22 | CARVYGVATNFGLYYYYYMDVW |
| ROME_291 | #5 | IGHV1-2 | IGHJ6 | | IGHD2-15 | unmutated | 22 | CAREEDVVVVAASYYYYGMDVW |
| ROME_356 | #5 | IGHV1-69 | IGHJ6 | | IGHD1-14 | unmutated | 22 | CARDEGYMWNHPYYYYYYMDVW |
| ROME_581 | #5 | IGHV1-69 | IGHJ6 | | IGHD3-10 | unmutated | 22 | CARSAVRGVIPGAYYYYGMDVW |
| ROME_587 | #5 | IGHV3-11 | IGHJ6 | | IGHD3-03 | unmutated | 22 | CARDLQSGYYQYYYYYYYMDVW |
| ROME_109 | #6 | IGHV1-69 | IGHJ3 | | IGHD3-16 | unmutated | 23 | CARGGDYDYVWGSYRPNDAFDIW |
| ROME_202 | #6 | IGHV1-69 | IGHJ3 | | IGHD3-16 | unmutated | 23 | CARGGVYDYVWGSYRSNDAFDIW |
| ROME_242 | #6 | IGHV1-69 | IGHJ3 | | IGHD3-16 | unmutated | 23 | CARGGGYDYIWGSYRPNDAFDIW |
| ROME_334 | #6 | IGHV1-69 | IGHJ6 | | IGHD3-16 | unmutated | 23 | CARGGYYDYVWGSYRPNDAFDIW |
| ROME_375 | #6 | IGHV1-69 | IGHJ3 | | IGHD3-16 | unmutated | 23 | CARGGEYDYVWGSYRTNDAFDIW |
| ROME_047 | #7B | IGHV4-4 | IGHJ6 | | IGHD3-3 | unmutated | 25 | CAREESYYDFWSGYRVDYYYYMDVW |
| ROME_404 | #7B | IGHV4-34 | IGHJ6 | | IGHD3-3 | unmutated | 25 | CARVNTYYDFWSGYNPFIYYYMDVW |
| ROME_532 | #7B | IGHV1-69 | IGHJ6 | | IGHD3-3 | unmutated | 25 | CARATFYYDFWSGYSPYYYYGMDVW |
| ROME_632 | #7B | IGHV1-69 | IGHJ6 | | IGHD3-3 | unmutated | 25 | CARATFYYDFWSGYSPYYYYGMDVW |
| ROME_093 | #7C | IGHV4-59 | IGHJ6 | | IGHD3-3 | unmutated | 26 | CARDTIAYDFWSGYLGPGVGYYMDVW |
| ROME_457 | #7C | IGHV1-69 | IGHJ6 | | IGHD3-3 | unmutated | 26 | CARGGPGVDFWSGYYPNYYYYGMDVW |
| ROME_618 | #7C | IGHV1-69 | IGHJ6 | | IGHD3-3 | unmutated | 26 | CARGAGERDFWSGYYPNYYYYGMDVW |
| ROME_621 | #7C | IGHV1-69 | IGHJ6 | | IGHD3-3 | unmutated | 26 | CARPAGDYDFWSGYYPNYYYYGMDVW |
| ROME_686 | #7C | IGHV1-69 | IGHJ6 | | IGHD3-3 | unmutated | 26 | CARDDGRYDFWSGYPNYYYYYYMDVW |
| ROME_736 | #7C | IGHV1-69 | IGHJ6 | | IGHD3-3 | unmutated | 26 | CARDGGDYDFWSGYYPNYYYYGMDVW |
| ROME_159 | #7D | IGHV1-69 | IGHJ6 | | IGHD3-3 | unmutated | 27 | CARDRGAYDFWSGYYDPRHYYYYMDVW |
| ROME_275 | #7D | IGHV3-48 | IGHJ6 | | IGHD3-3 | unmutated | 27 | CARDYVWNYDFWSGYYTDYYYYGMDVW |
| ROME_303 | #7D | IGHV1-69 | IGHJ6 | | IGHD3-3 | unmutated | 27 | CARDGTTQYDFWSGYYPNYYYYGMDVW |
| ROME_092 | #7E | IGHV1-69 | IGHJ6 | | IGHD3-3 | unmutated | 28 | CARPRGGEYYDFWSGYYPNYYYYGMDVW |
| ROME_198 | #7E | IGHV1-69 | IGHJ6 | | IGHD3-3 | unmutated | 28 | CARGGIIWDFWSGYVVPDYYYYYGMDVW |
| ROME_671 | #7F | IGHV1-69 | IGHJ6 | | IGHD3-3 | unmutated | 29 | CARADPGYDFWSGYSLGPFLYYYYGMDVW |
| ROME_078 | #8 | IGHV4-39 | IGHJ5 | | IGHD6-13 | unmutated | 20 | CAKSTGYSSSWYSNTWFDPW |
| ROME_141 | #8 | IGHV4-39 | IGHJ5 | | IGHD6-13 | unmutated | 20 | CAQSTGYSSSWYSNRWFDPW |
| ROME_216 | #8 | IGHV4-39 | IGHJ5 | | IGHD6-19 | unmutated | 21 | CARQYGYSSGWYGRANWFDPW |
| ROME_311 | #8 | IGHV4-39 | IGHJ5 | | IGHD6-13 | unmutated | 21 | CARHVGYSSSWYNVFSWFDPW |
| ROME_614 | #8 | IGHV4-39 | IGHJ5 | | IGHD6-13 | unmutated | 20 | CATATGYSSSWYGVNWFDPW |
| ROME_322 | #9 | IGHV1-69 | IGHJ6 | | IGHD3-3 | unmutated | 25 | CARVGGITIFGVVIQTHYYYYMDVW |
| ROME_743 | #9 | IGHV1-69 | IGHJ6 | | IGHD3-3 | unmutated | 22 | CARDLGVTIFGVVITPYGMDVW |
| ROME_072 | #10 | IGHV1-46 | IGHJ6 | | IGHD2-2 | unmutated | 25 | CARDGGYCSSTSCYISIDYYGMDVW |
| ROME_227 | #10 | IGHV1-69 | IGHJ6 | | IGHD2-2 | unmutated | 27 | CARVGAGYCSSTSCYPDYYYYYGMDVW |
| ROME_777 | #10 | IGHV4-39 | IGHJ6 | | IGHD2-2 | unmutated | 24 | CARHRLGYCSSTSCYYYYYGMDVW |
| ROME_260 | #12 | IGHV1-2 | IGHJ4 | | IGHD3-22 | unmutated | 21 | CARDGYYYDSSGYYSYYFDYW |
| ROME_524 | #12 | IGHV1-46 | IGHJ4 | | IGHD3-22 | unmutated | 21 | CARDQYYYDSSGYYSGYFDYW |
| ROME_590 | #14 | IGHV3-7 | IGHJ4 | | IGHD3-9 | mutated | 13 | CARGFRWLQFDSW |
| ROME_056 | #16 | IGHV4-34 | IGHJ6 | | IGHD2-15 | mutated | 26 | CAGRFYCSGGSCSREDLYFYQGLDAW |
| ROME_207 | #16 | IGHV4-34 | IGHJ6 | | IGHD6-19 | mutated | 26 | CAGRFYCSGITCSRVEFYHYYGMDVW |
| ROME_213 | #16 | IGHV4-34 | IGHJ6 | | IGHD2-15 | mutated | 26 | CAGRFYCSGETCSSPSFYYYYGMDVW |
| ROME_434 | #16 | IGHV4-34 | IGHJ6 | | IGHD2-15 | mutated | 26 | CAGRFYCAGDTCYSAAFHYYSGLDVW |
| ROME_501 | #16 | IGHV4-34 | IGHJ6 | | IGHD2-15 | mutated | 26 | CAREFYCNGASCARPDYYYYSGMDVW |
| ROME_533 | #20 | IGHV3-53 | IGHJ4 | | IGHD4-23 | unmutated | 13 | CSGGNSPGLFDYW |
| ROME_020 | #21 | IGHV1-69 | IGHJ6 | | IGHD3-22 | unmutated | 25 | CARVVPYDSSGYSIFYYYYYGMDVW |
| ROME_352 | #21 | IGHV3-30 | IGHJ6 | | IGHD3-3 | unmutated | 30 | CARDGKGSRVYYDFWSAPPPYYYYYYMDVW |
| ROME_298 | #22 | IGHV1-2 | IGHJ6 | | IGHD3-3 | unmutated | 9 | CARDEAAYYDFWSGYYGMDVW |
| ROME_519 | #22 | IGHV1-69 | IGHJ6 | | IGHD3-3 | unmutated | 9 | CARADGYDFWSGPLYYYYYMDVW |
| ROME_203 | #25 | IGHV3-33 | IGHJ6 | | IGHD3-3 | unmutated | 9 | CAKDLVLRFLEWPLYYYYYGMDVW |
| ROME_230 | #25 | IGHV1-8 | IGHJ6 | | IGHD3-3 | unmutated | 9 | CARGQLLRFLEWLVDYYYGMDVW |
| ROME_785 | #25 | IGHV3-21 | IGHJ6 | | IGHD3-3 | unmutated | 9 | CARDLGFLEWFGGSDYYYYGMDVW |
| ROME_780 | #26 | IGHV1-69 | IGHJ6 | | IGHD6-13 | unmutated | 9 | CARDSSSWYRRGWEYYYGMDVW |
| ROME_317 | #28A | IGHV1-2 | IGHJ6 | | IGHD1-26 | unmutated | 19 | CARMMCGSYYYYYYGMDVW |
| ROME_531 | #28A | IGHV1-2 | IGHJ6 | | IGHD1-26 | unmutated | 19 | CARLYSGSYYYYYYGMDVW |
| ROME_710 | #28A | IGHV3-53 | IGHJ6 | | IGHD3-3 | unmutated | 19 | CARVLTTFGVYYYYGMDVW |
| ROME_394 | #29 | IGHV4-34 | IGHJ3 | | IGHD6-19 | mutated | 7 | CARDIAVAPPDAFDVW |
| ROME_771 | #29 | IGHV4-34 | IGHJ3 | | IGHD-6-19 | mutated | 7 | CARDIAVPPPDAFDIW |
| ROME_376 | #30 | IGHV3-9 | IGHJ4 | | IGHD3-3 | unmutated | 9 | CAKDYYDFWSGYPNDSPFDYW |
| ROME_726 | #30 | IGHV3-9 | IGHJ4 | | IGHD3-3 | unmutated | 9 | CAKDFYDFWSGYPNFSPFDYW |
| ROME_198 | #31 | IGHV3-7 | IGHJ6 | | IGHD3-22 | unmutated | 23 | CARVTYDSSGPQYYYYYYGMDVW |
| ROME_246 | #31 | IGHV3-48 | IGHJ6 | | IGHD3-3 | unmutated | 23 | CARDYDFWSGYYAYYYYYGMDVW |
| ROME_264 | #31 | IGHV3-48 | IGHJ6 | | IGHD3-3 | unmutated | 23 | CARDYDFWSGYYSYYYYYGMDVW |
| ROME_747 | #31 | IGHV3-33 | IGHJ6 | | IGHD3-9 | unmutated | 22 | CARDPDYDILTGPYYYYGMDVW |
| ROME_691 | #32 | IGHV3-11 | IGHJ6 | | IGHD3-22 | unmutated | 26 | CARAPKYYYDSSGYWWNYYYYGMDVW |
| ROME_026 | #35 | IGHV1-2 | IGHJ6 | | IGHD3-22 | unmutated | 27 | CARGGGGVDSSGYYYDIDYYYYGMDVW |
| ROME_390 | #35 | IGHV4-59 | IGHJ6 | | IGHD3-22 | unmutated | 28 | CARAIGGDYYDSSGYYYVNYYYYGMDVW |
| ROME_325 | #37 | IGHV4-39 | IGHJ4 | | IGHD6-19 | unmutated | 17 | CARSRYSSGWYDYFDYW |
| ROME_131 | #38 | IGHV1-69 | IGHJ5 | | IGHD3-3 | unmutated | 22 | CARDGGDYDFWSGYYRRWFDPW |
| ROME_675 | #38 | IGHV1-18 | IGHJ5 | | IGHD3-3 | unmutated | 23 | CARDPYYDFWSGAAKTYNWFDPW |
| ROME_029 | #40 | IGHV3-30 | IGHJ4 | | IGHD3-9 | mutated | 16 | CARGIVGTIDATFDYW |
| ROME_189 | #40 | IGHV3-30 | IGHJ4 | | IGHD1-26 | mutated | 16 | CTRGIVGTTDDTFDFW |
| ROME_285 | #40 | IGHV2-5 | IGHJ4 | | IGHD1-7 | unmutated | 15 | CAHEGITGTTGFDYW |
| ROME_362 | #40 | IGHV3-30 | IGHJ4 | | IGHD1-26 | mutated | 16 | CARGIVGTTDGVFDYW |
| ROME_055 | #50 | IGHV4-59 | IGHJ6 | | IGHD3-22 | unmutated | 24 | CARGRGDYYDSSGYLRYYYGMDVW |
| ROME_440 | #50 | IGHV3-33 | IGHJ6 | | IGHD3-22 | unmutated | 24 | CAGGGDYYDSSGYFYYYYYGMDVW |
| ROME_693 | #50 | IGHV3-20 | IGHJ6 | | IGHD3-22 | unmutated | 25 | CARAAYYYDSSGYYQRGYYYGMDVW |
| ROME_327 | #53 | IGHV1-2 | IGHJ4 | | IGHD3-10 | mutated | 16 | CASWGGYGSGSYFDYW |
| ROME_544 | #57 | IGHV1-69 | IGHJ6 | | IGHD3-16 | unmutated | 25 | CARGHDYIWGSYRSPDYYYYYMDVW |
| ROME_083 | #59 | IGHV1-69 | IGHJ6 | | IGHD3-3 | unmutated | 13 | CARAYDFWSGYSW |
| ROME_199 | #59 | IGHV1-69 | IGHJ4 | | IGHD3-3 | unmutated | 14 | CARGYDFWSGYHTW |
| ROME_413 | #59 | IGHV1-58 | IGHJ6 | | IGHD3-3 | unmutated | 14 | CAAGKDFWSGLDVW |
| ROME_423 | #59 | IGHV1-69 | IGHJ5 | | IGHD3-3 | unmutated | 14 | CAMGYDFWSGYNYW |
| ROME_598 | #64B | IGHV4-34 | IGHJ6 | | IGHD2-2 | unmutated | 23 | CARGDLLVVPAAIYYYYYGMDVW |
| ROME_709 | #64B | IGHV3-30 | IGHJ6 | | IGHD2-15 | unmutated | 23 | CAREELLVVVAAIYYYYYGMDVW |
| ROME_753 | #64B | IGHV3-30 | IGHJ6 | | IGHD2-15 | unmutated | 23 | CAREELLVVVAAIYYYYYGMDVW |
| ROME_757 | #64B | IGHV3-48 | IGHJ6 | | IGHD2-2 | unmutated | 23 | CARESPLVVPAAIFYYYYGMDVW |
| ROME_075 | #67 | IGHV1-2 | IGHJ4 | | IGHD3-3 | mutated | 17 | CARGITMAGNGGDFDYW |
| ROME_123 | #67 | IGHV1-2 | IGHJ5 | | IGHD2-21 | mutated | 17 | CARGVTVTGDEGDFDCW |
| ROME_302 | #70 | IGHV3-23 | IGHJ4 | | IGHD3-10 | unmutated | 16 | CAKDPGILWFGEVEYW |
| ROME_737 | #71 | IGHV5-10-1 | IGHJ6 | | IGHD6-13 | unmutated | 24 | CARHDSIAAAGTWDYYYYYGMDVW |
| ROME_760 | #71 | IGHV5-10-1 | IGHJ6 | | IGHD6-13 | unmutated | 24 | CARHWGIAAAGTWDYYYYYGMDVW |
| ROME_149 | #75 | IGHV4-34 | IGHJ6 | | IGHD2-2 | unmutated | 28 | CARGDRRTGYCSSTSCYLYYYYYGMDVW |
| ROME_589 | #75 | IGHV4-34 | IGHJ6 | | IGHD2-2 | unmutated | 28 | CARGMRSLGYCSSTSCYLYYYYYGMDVW |
| ROME_324 | #77 | IGHV4-59 | IGHJ4 | | IGHD6-19 | mutated | 16 | CARGPHMSGWNAFEYW |
| ROME_419 | #77 | IGHV4-4 | IGHJ1 | | IGHD6-19 | mutated | 16 | CTRGADSSGWHSFQYW |
| ROME_361 | #79 | IGHV3-30-3 | IGHJ6 | | IGHD2-2 | unmutated | 25 | CASVSLLPVVVPAAINYYYYGMDVW |
| ROME_344 | #89 | IGHV4-30-4 | IGHJ4 | | IGHD3-22 | unmutated | 23 | CARTRGYYDSSGYYYTLTPFDYW |
| ROME_435 | #89 | IGHV4-61 | IGHJ3 | | IGHD3-22 | unmutated | 23 | CARDLLYYYDSSGYYSDDAFDIW |
| ROME_569 | #90 | IGHV3-9 | IGHJ4 | | IGHD3-22 | mutated | 15 | CAKDGGSSGYYVDYW |
| ROME_190 | #95 | IGHV1-46 | IGHJ4 | | IGHD3-22 | unmutated | 17 | CARSPWSSGYYSYFDYW |
| ROME_573 | #95 | IGHV3-30 | IGHJ4 | | IGHD3-22 | mutated | 15 | CARSYDSSGYYLVYW |
| ROME_086 | #96 | IGHV5-51 | IGHJ4 | | IGHD3-3 | unmutated | 23 | CARHQTYYDFWSGYLLPGALDYW |
| ROME_279 | #98 | IGHV3-7 | IGHJ6 | | IGHD3-3 | unmutated | 25 | CARGGTTYYDFWSGPFYYYYGMDVW |
| ROME_666 | #98 | IGHV3-9 | IGHJ6 | | IGHD3-3 | unmutated | 26 | CAKDFRAYYDFWSGYSAYYYYGMDVW |
| ROME_034 | #99 | IGHV1-3 | IGHJ4 | | IGHD6-19 | unmutated | 16 | CARVQWLALGDYFDYW |
| ROME_249 | #99 | IGHV1-46 | IGHJ4 | | IGHD6-19 | unmutated | 16 | CARAQWLGYSPNFDYW |
| ROME_572 | #99 | IGHV7-81 | IGHJ4 | | IGHD6-19 | unmutated | 16 | CARLQWLGPVENFDYW |
| ROME_752 | #99 | IGHV1-2 | IGHJ4 | | IGHD6-19 | unmutated | 16 | CAREQWLALGYYFDYW |
| ROME_119 | #109 | IGHV3-11 | IGHJ5 | | IGHD3-10 | unmutated | 21 | CARESCLYYGSGSYYNWFDPW |
| ROME_177 | #109 | IGHV3-11 | IGHJ5 | | IGHD3-10 | unmutated | 21 | CARERILYYGSGSYFNWFDPW |
| ROME_235 | #109 | IGHV3-11 | IGHJ5 | | IGHD3-10 | unmutated | 21 | CAREEVLYYGSGSYFNWFDPW |
| ROME_707 | #117 | IGHV5-51 | IGHJ4 | | IGHD3-22 | unmutated | 17 | CARPQGSSGYYGYFDYW |
| ROME_508 | #201 | IGHV4-34 | IGHJ3 | | IGHD3-22 | mutated | 18 | CARREFYDRRGNDGFDMW |
| ROME_006 | #202 | IGHV3-48 | IGHJ4 | | IGHD5-24 | mutated | 16 | CARGRDGYNLAPIDYW |
| ROME_091 | #202 | IGHV3-30 | IGHJ4 | | IGHD1-26 | mutated | 16 | CARGIVGATDGVFDYW |
| ROME_228 | #202 | IGHV3-33 | IGHJ3 | | IGHD3-16 | unmutated | 16 | CARGTRGDYTWAFDIW |
| ROME_412 | #202 | IGHV3-30-3 | IGHJ3 | | IGHD4-17 | unmutated | 16 | CARGPKGDYLHAFDIW |
| ROME_473 | #202 | IGHV3-30 | IGHJ4 | | IGHD4-17 | unmutated | 16 | CARGPRGDYVFPFDYW |
| ROME_593 | #202 | IGHV6-1 | IGHJ3 | | IGHD3-16 | Mutated | 16 | CARGGYGVSVFLFDFW |
| ROME_784 | #202 | IGHV3-23 | IGHJ4 | | IGHD4-17 | mutated | 16 | CAKGDSGDSPSSFDYW |
| ROME_585 | #203 | IGHV1-69 | IGHJ6 | | IGHD5-18 | unmutated | 22 | CIRTRPKYSYGYDYYYYGMDVW |
| ROME_660 | #207 | IGHV4-59 | IGHJ4 | | IGHD6-19 | mutated | 16 | CARDRSSVFPGTYDFW |
| ROME_482 | #211 | IGHV3-21 | IGHJ4 | | IGHD3-09 | unmutated | 22 | CARVSTEYDILTAYYSYYFDYW |
| ROME_004 | #217 | IGHV1-69 | IGHJ4 | | IGHD2-2 | unmutated | 21 | CARDQGPYCSSTSCYGYFDYW |
| ROME_570 | #220 | IGHV3-7 | IGHJ3 | | IGHD3-22 | mutated | 20 | CARGDYYDSSGYMHDAFDIW |
| ROME_329 | #222 | IGHV3-30 | IGHJ6 | | IGHD6-19 | mutated | 21 | CAKVTLAGSFEAFHYYGMDVW |
| ROME_054 | #224 | IGHV4-34 | IGHJ3 | | IGHD5-18 | mutated | 16 | CARDGWLRPPDAFDIW |
| ROME_087 | #226 | IGHV3-7 | IGHJ4 | | IGHD2-2 | mutated | 20 | CARTYCSSNTCYPDRLLDYW |
| ROME_490 | #229 | IGHV3-23 | IGHJ6 | | IGHD2-2 | unmutated | 27 | CAKGRDCSSTSCYIGMNYYYYYGMDVW |
| ROME_082 | #230 | IGHV1-69 | IGHJ6 | | IGHD3-9 | unmutated | 24 | CASSLYYDILTGYYYYYYYGMDVW |
| ROME_156 | #235 | IGHV4-39 | IGHJ6 | | IGHD6-19 | unmutated | 20 | CARRGQWLDNYYYYYGMDVW |
| ROME_723 | #242 | IGHV4-39 | IGHJ4 | | IGHD3-22 | unmutated | 20 | CARLRWYYYDSSGYAPFDYW |
| ROME_238 | #148B | IGHV2-5 | IGHJ4 | | IGHD2-2 | mutated | 20 | CAHRRVGSSNTWDSGLFDYW |
| ROME_300 | #148B | IGHV2-5 | IGHJ4 | | IGHD3-9 | mutated | 19 | CAHRGTLTGFWDSGYFDYW |
| ROME_460 | #NEW1_ROME | IGHV1-69 | IGHJ5 | | IGHD2-2 | unmutated | 14 | CARRQLLFYWFDPW |
| ROME_596 | #NEW1_ROME | IGHV1-69 | IGHJ5 | | IGHD2-2 | unmutated | 14 | CARRQLLYYWFDPW |
| ROME_169 | #NEW2_ROME | IGHV3-11 | IGHJ6 | | IGHD5-24 | unmutated | 20 | CAREPRWLQSNYYYYGMDVW |
| ROME_603 | #NEW2_ROME | IGHV3-11 | IGHJ6 | | IGHD5-24 | unmutated | 20 | CAREPRWLQSNYYYYGMDVW |
| ROME_353 | #NEW3_ROME | IGHV1-69 | IGHJ6 | | IGHD3-10 | unmutated | 21 | CASRAISYYGSGIDYYGMDVW |
| ROME_540 | #NEW3_ROME | IGHV1-69 | IGHJ6 | | IGHD3-10 | unmutated | 21 | CASRAISYYGSGIDYYGMDVW |
| ROME_400 | #NEW4_ROME | IGHV4-61 | IGHJ3 | | IGHD7-27 | mutated | 16 | CSTTGGPPKAHAFDVW |
| ROME_452 | #NEW4_ROME | IGHV4-30-2 | IGHJ3 | | IGHD7-27 | mutated | 16 | CSTTGGPPKAHAFDVW |
| ROME_136 | #NOVEL 1 | IGHV4-59 | IGHJ6 | | IGHD3-22 | unmutated | 24 | CARGDYYDSSGYYYLGNYYYMDVW |
| ROME_767 | #NOVEL 1 | IGHV4-59 | IGHJ6 | | IGHD3-22 | unmutated | 25 | CARGDYYDSSGYYYVGYYYYYMDVW |
