# Supplementary material for: Immunoglobulin gene rearrangements in Chinese and Italian patients with chronic lymphocytic leukemia
Source: Oncotarget. 2016 Mar 1;7(15):20520–31. doi: 10.18632/oncotarget.7819 (PMC4991472; doi:10.18632/oncotarget.7819)
Supplement: Supplementary file 3 [file oncotarget-07-20520-s003.doc]

Supplementary Table S2A: Chinese IGHV Mutational status

| Mutational status | Nanjing No | Hong Kong No | | Tianjin No | Chinese No | % |
| --- | --- | --- | --- | --- | --- | --- |
| M | 209 | 81 | 125 | | 415 | 65,8 |
| UM | 136 | 39 | 41 | | 216 | 34,2 |
| totals | 345 | 120 | 166 | | 631 | 100,0 |


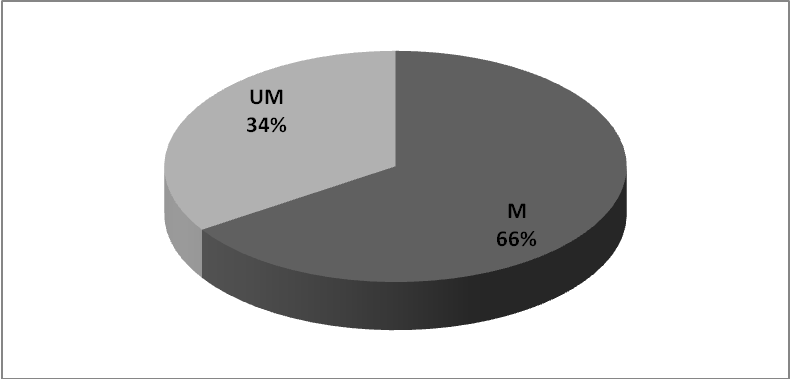


Supplementary Table S2B: Chinese IGHV subgroup and gene repertoire.

| IGHV Subgroup | Chinese No | % |
| --- | --- | --- |
| IGHV1 | 98 | 15,5 |
| IGHV2 | 21 | 3,3 |
| IGHV3 | 295 | 46,8 |
| IGHV4 | 181 | 28,7 |
| IGHV5 | 15 | 2,4 |
| IGHV6 | 10 | 1,6 |
| IGHV7 | 11 | 1,7 |
| totals | 631 | 100,0 |


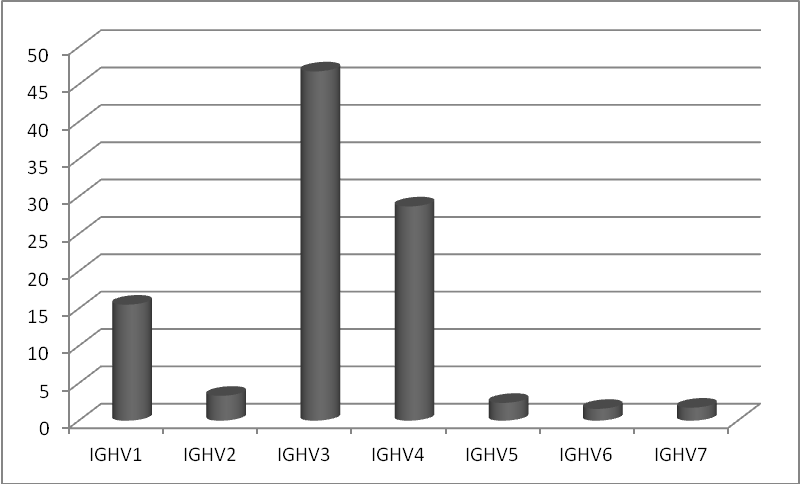


| IGHV gene | Chinese No | % |
| --- | --- | --- |
| V1-18 | 14 | 2,2 |
| V1-2 | 11 | 1,7 |
| V1-24 | 1 | 0,2 |
| V1-3 | 23 | 3,7 |
| V1-46 | 4 | 0,6 |
| V1-58 | 2 | 0,3 |
| V1-69 | 36 | 5,7 |
| V1-8 | 7 | 1,1 |
| V2-26 | 1 | 0,2 |
| V2-5 | 16 | 2,5 |
| V2-70 | 4 | 0,6 |
| V3-11 | 14 | 2,2 |
| V3-13 | 3 | 0,5 |
| V3-15 | 14 | 2,2 |
| V3-20 | 6 | 1,0 |
| V3-21 | 19 | 3,0 |
| V3-23 | 53 | 8,4 |
| V3-30 | 25 | 4,0 |
| V3-30-3 | 7 | 1,1 |
| V3-33 | 18 | 2,9 |
| V3-35 | 0 | 0,0 |
| V3-43 | 2 | 0,3 |
| V3-48 | 22 | 3,5 |
| V3-49 | 5 | 0,8 |
| V3-53 | 9 | 1,4 |
| V3-64 | 2 | 0,3 |
| V3-66 | 4 | 0,6 |
| V3-7 | 51 | 8,1 |
| V3-72 | 4 | 0,6 |
| V3-73 | 3 | 0,5 |
| V3-74 | 23 | 3,7 |
| V3-9 | 11 | 1,7 |
| V4-30-2 | 4 | 0,6 |
| V4-30-4 | 2 | 0,3 |
| V4-31 | 7 | 1,1 |
| V4-34 | 70 | 11,1 |
| V4-38-2 | 3 | 0,5 |
| V4-39 | 43 | 6,8 |
| V4-4 | 12 | 1,9 |
| V4-59 | 30 | 4,8 |
| V4-61 | 10 | 1,6 |
| V5-10-1 | 2 | 0,3 |
| V5-51 | 13 | 2,1 |
| V6-1 | 10 | 1,6 |
| V7-4-1 | 11 | 1,7 |
| V7-81 | 0 | 0,0 |
| totals | 631 | 100,0 |


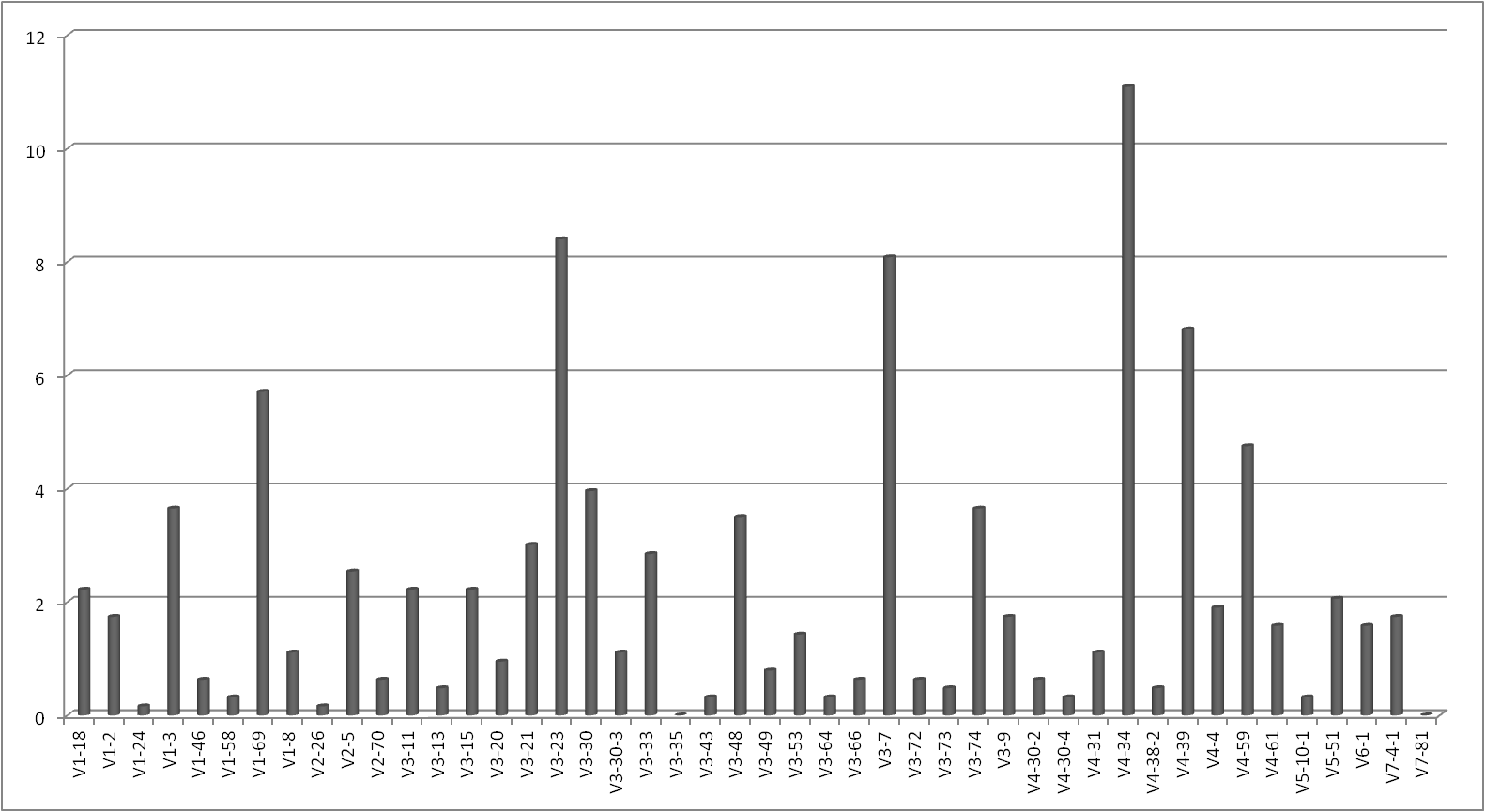


Supplementary Table S2C: Chinese IGHD subgroup and gene repertoire

| IGHD Subgroup | Chinese No | % |
| --- | --- | --- |
| IGHD1 | 62 | 10,0 |
| IGHD2 | 91 | 14,6 |
| IGHD3 | 239 | 38,4 |
| IGHD4 | 38 | 6,1 |
| IGHD5 | 62 | 10,0 |
| IGHD6 | 122 | 19,6 |
| IGHD7 | 8 | 1,3 |
| totals | 622 | 100,0 |


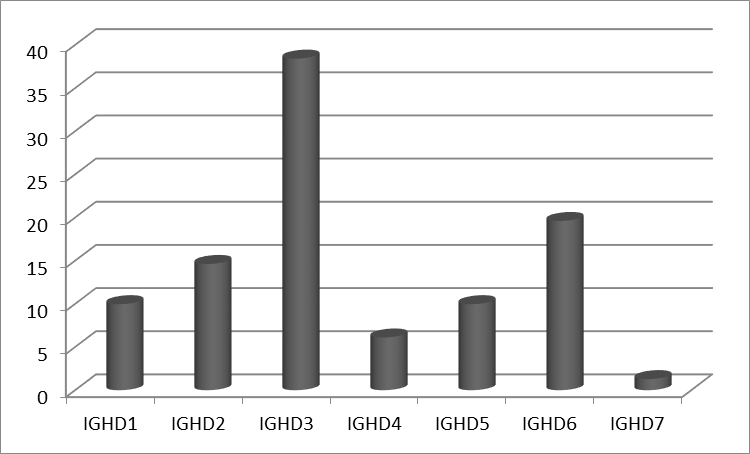


| IGHD gene | Chinese No | % |
| --- | --- | --- |
| IGHD1-1 | 13 | 2,1 |
| IGHD1-14 | 2 | 0,3 |
| IGHD1-20 | 3 | 0,5 |
| IGHD1-21 | 1 | 0,2 |
| IGHD1-26 | 35 | 5,6 |
| IGHD1-7 | 8 | 1,3 |
| IGHD2-15 | 17 | 2,7 |
| IGHD2-2 | 38 | 6,1 |
| IGHD2-21 | 28 | 4,5 |
| IGHD2-8 | 8 | 1,3 |
| IGHD3-10 | 74 | 11,9 |
| IGHD3-16 | 34 | 5,5 |
| IGHD3-22 | 59 | 9,5 |
| IGHD3-3 | 45 | 7,2 |
| IGHD3-9 | 27 | 4,3 |
| IGHD4-11 | 2 | 0,3 |
| IGHD4-17 | 21 | 3,4 |
| IGHD4-23 | 14 | 2,3 |
| IGHD4-4 | 1 | 0,2 |
| IGHD5-12 | 31 | 5,0 |
| IGHD5-18 | 13 | 2,1 |
| IGHD5-24 | 10 | 1,6 |
| IGHD5-5 | 8 | 1,3 |
| IGHD6-13 | 54 | 8,7 |
| IGHD6-19 | 53 | 8,5 |
| IGHD6-25 | 2 | 0,3 |
| IGHD6-6 | 13 | 2,1 |
| IGHD7-27 | 8 | 1,3 |
| totals | 622 | 100,0 |


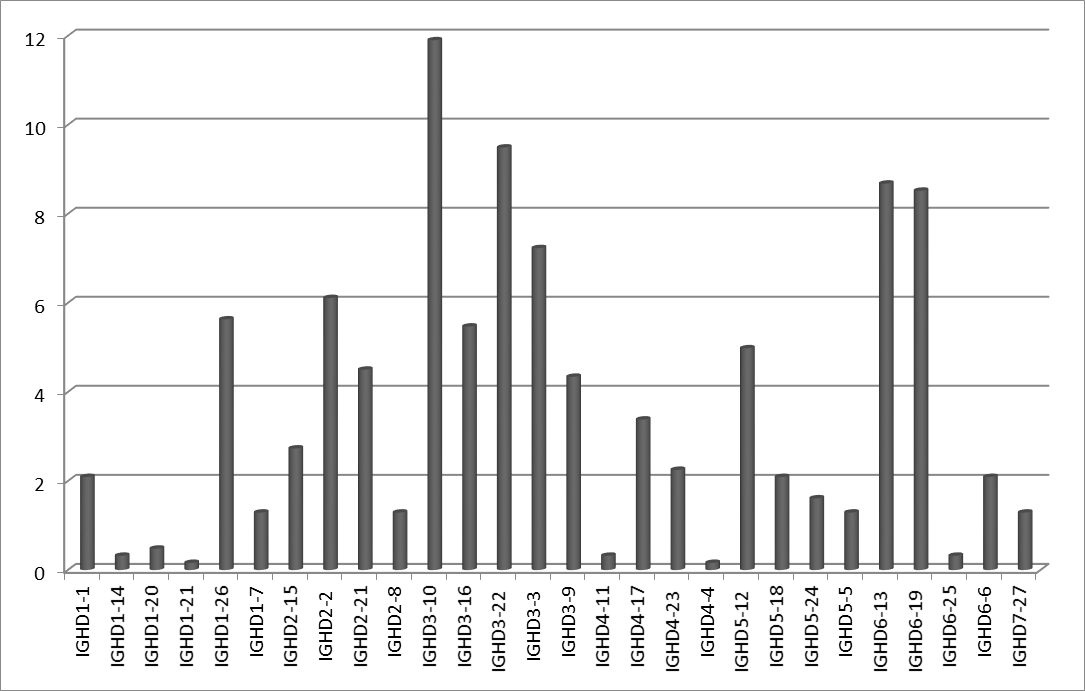


Supplementary Table S2D: Chinese IGHJ gene repertoire

| IGHJ gene | | Chinese No | | % |
| --- | --- | --- | --- | --- |
| IGHJ1 | 12 | | 1,9 | |
| IGHJ2 | 8 | | 1,3 | |
| IGHJ3 | 68 | | 10,8 | |
| IGHJ4 | 289 | | 45,8 | |
| IGHJ5 | 93 | | 14,7 | |
| IGHJ6 | 161 | | 25,5 | |
| totals | 631 | | 100,0 | |


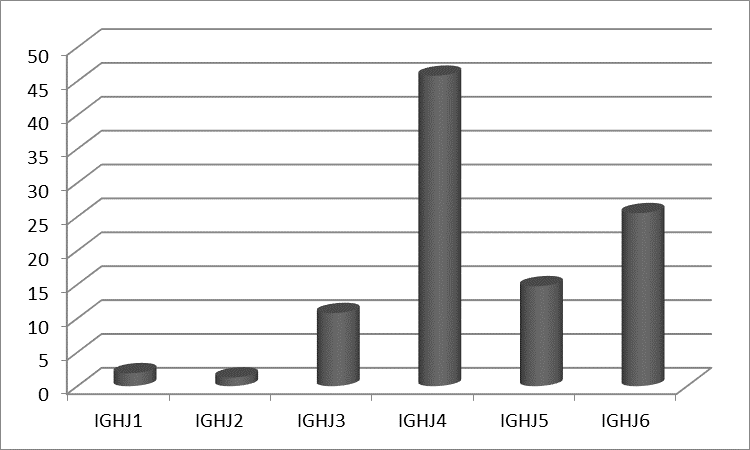


Supplementary Table S2E: Chinese Stereotyped and Non-Stereotyped BCRs and Major Subsets

| Chinese CLL | No | % |
| --- | --- | --- |
| Stereotyped BCRs | 124 | 19,7 |
| Non Stereotyped BCRs | 507 | 80,3 |
| totals | 631 | 100 |


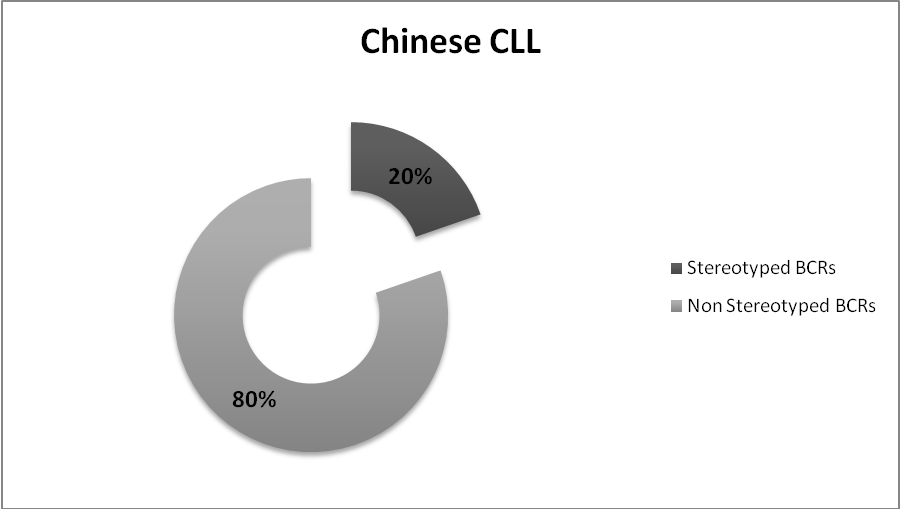


| Major Subset | IGHV gene(s) | No | % |
| --- | --- | --- | --- |
| #8 | V4-39 | 18 | 27,3 |
| #1 | Clan I V genes | 14 | 21,2 |
| #4 | V4-34 | 11 | 16,7 |
| #5 | V1-69 | 4 | 6,1 |
| #6 | V1-69 | 4 | 6,1 |
| #77 | V4 (V4-4, V4-59) | 3 | 4,5 |
| #3 | V1-69 | 2 | 3 |
| #28a | V1 (V1-2) | 2 | 3 |
| #31 | Clan III V genes | 2 | 3 |
| #99 | Clan I V genes | 2 | 3 |
| #2 | V3-21 | 1 | 1,5 |
| #12 | V1-2, V1-46 | 1 | 1,5 |
| #16 | V4-34 | 1 | 1,5 |
| #59 | Clan I V genes | 1 | 1,5 |
| totals |  | 66 | 100 |


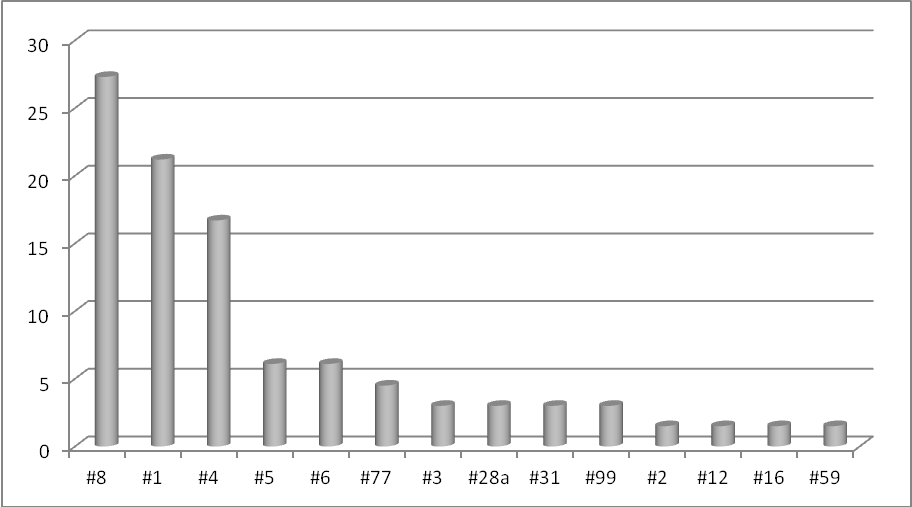


Supplementary Table S2F: Characteristics of the Chinese subsets

| **Sample** | **Subset No** | **IGHV gene(s)** | **IGHD gene(s)** | **IGHJ gene(s)** | **Mutational status** | **VH CDR3 length** | **HCDR3 AMINOACIDS** |
| --- | --- | --- | --- | --- | --- | --- | --- |
| HK_43 | #1 | IGHV5-51 | IGHJ4 | IGHD3-10 | unmutated | 15 | CARHQWFGVNYFDYW |
| HK_114 | #1 | IGHV5-51 | IGHJ4 | IGHD3-10 | unmutated | 15 | CARHLWLGEYYFDYW |
| HK_122 | #1 | IGHV1-3 | IGHJ4 | IGHD6-19 | unmutated | 15 | CAREQWLGILNIDYW |
| NJ_24 | #1 | IGHV1-3 | IGHJ4 | IGHD6-19 | unmutated | 15 | CARKQWLGPPYFDYW |
| NJ_55 | #1 | IGHV1-3 | IGHJ4 | IGHD6-19 | unmutated | 15 | CARGQWLVLLSFDYW |
| NJ_225 | #1 | IGHV1-18 | IGHJ4 | IGHD6-19 | unmutated | 15 | CARYQWLGSYYFDYW |
| NJ_305 | #1 | IGHV5-10-1 | IGHJ4 | IGHD6-19 | unmutated | 15 | CARQQWLEGEYFDYW |
| NJ_308 | #1 | IGHV5-51 | IGHJ4 | IGHD3-10 | unmutated | 15 | CARHLWFGEYYFDYW |
| NJ_380 | #1 | IGHV1-2 | IGHJ4 | IGHD6-19 | unmutated | 15 | CARQQWLVLDYFDYW |
| NJ_382 | #1 | IGHV1-3 | IGHJ4 | IGHD6-19 | unmutated | 15 | CAREQWLVNPNFDYW |
| NJ_384 | #1 | IGHV1-3 | IGHJ4 | IGHD6-19 | unmutated | 15 | CAREQWLPLYYFDYW |
| NJ_10 | #1 | IGHV1-3 | IGHJ4 | IGHD6-19 | unmutated | 15 | CAREQWLVNPNFDYW |
| TJ_04 | #1 | IGHV5-10-1 | IGHJ4 | IGHD6-19 | mutated | 15 | CAREQWLVRTHFDYW |
| TJ_166 | #1 | IGHV1-3 | IGHJ4 | IGHD2-21 | unmutated | 15 | CARDQWWLLTYFDYW |
| NJ_153 | #2 | IGHV3-30-3 | IGHJ6 | IGHD5-5 | mutated | 11 | CARDSYGMDVW |
| NJ_90 | #3 | IGHV1-69 | IGHJ6 | IGHD2-2 | unmutated | 24 | CARDCPDIVVVPAAIRRYYGMDVW |
| TJ_21 | #3 | IGHV1-69 | IGHJ6 | IGHD2-2 | unmutated | 24 | CARDVPDIVVVPAAITYYYGMDVW |
| HK_03 | #4 | IGHV6-1 | IGHJ6 | IGHD2-21 | mutated | 22 | CARTRGMGVGTARDYYYGMDVW |
| NJ_21 | #4 | IGHV4-34 | IGHJ6 | IGHD1-1 | mutated | 22 | CARGYGDNWTSRRYYYYGMDVW |
| NJ_140 | #4 | IGHV4-34 | IGHJ6 | IGHD1-26 | mutated | 22 | CARGYGVDPTTRRYYYYGMDVW |
| NJ_208 | #4 | IGHV4-34 | IGHJ6 | IGHD2-21 | mutated | 22 | CSRGYGSTPVIRRYYYNGMDVW |
| NJ_411 | #4 | IGHV4-34 | IGHJ6 | IGHD4-17 | mutated | 22 | CARGYGDTEVTRRYYYYGMDVW |
| NJ_417 | #4 | IGHV4-34 | IGHJ6 | IGHD5-18 | mutated | 22 | CARGYPDTAVVKRYYFYGMDVW |
| NJ_424 | #4 | IGHV4-34 | IGHJ6 | IGHD5-18 | mutated | 22 | CTRGYPDTPMVRRYYHYVMDVW |
| TJ_35 | #4 | IGHV4-34 | IGHJ6 | IGHD5-18 | unmutated | 22 | CARGYGDSSVAKRYYFYGMDVW |
| TJ_109 | #4 | IGHV4-34 | IGHJ6 | IGHD1-26 | mutated | 22 | CATGYGVGPTLRRYYYYGMDVW |
| TJ_118 | #4 | IGHV4-34 | IGHJ6 | IGHD4-17 | mutated | 22 | CARGYGVTPVTRRYYYYGMDVW |
| TJ_152 | #4 | IGHV4-34 | IGHJ6 | IGHD5-18 | unmutated | 22 | CARGYGDSSVAKRYYFYGMDVW |
| HK_13 | #5 | IGHV3-74 | IGHJ6 | IGHD6-13 | unmutated | 22 | CARGRSIAAAVIYYYYYYMDVW |
| HK_71 | #5 | IGHV1-69 | IGHJ6 | IGHD5-18 | unmutated | 23 | CARARIEGVVQLWLTYYYGMDVW |
| HK_99 | #5 | IGHV1-69 | IGHJ6 | IGHD1-26 | unmutated | 22 | CARGRYSGSYYEYYYYYGMDVW |
| TJ_36 | #5 | IGHV3-30 | IGHJ6 | IGHD1-7 | mutated | 22 | CAREGITGTTVLNYYYYYMDVW |
| NJ_207 | #6 | IGHV1-69 | IGHJ3 | IGHD3-16 | unmutated | 23 | CARGGDYDYVWGSYRPNDAFDIW |
| NJ_448 | #6 | IGHV1-69 | IGHJ3 | IGHD3-16 | unmutated | 23 | CARGGPYDYVWGSYRPNDAFDIW |
| TJ_55 | #6 | IGHV1-69 | IGHJ3 | IGHD3-16 | mutated | 23 | CARGGDYDYVWGSYRPNDAFDIW |
| TJ_57 | #6 | IGHV1-69 | IGHJ3 | IGHD3-16 | mutated | 23 | CARGGDYDYIWGSYRTNDAFDIW |
| NJ_182 | #7B | IGHV1-69 | IGHJ6 | IGHD3-3 | unmutated | 25 | CASEGGYDFWSGYYPNYYYYGMDVW |
| NJ_392 | #7B | IGHV4-34 | IGHJ6 | IGHD3-3 | unmutated | 25 | CARASTYYDFWSGYRPHYYYYMDVW |
| NJ_407 | #7B | IGHV4-30-2 | IGHJ6 | IGHD3-3 | unmutated | 25 | CARGTQYYDFWSGYNPLYYYYMDVW |
| NJ_440 | #7D | IGHV1-69 | IGHJ6 | IGHD3-3 | unmutated | 27 | CARGSPNYDFWSGYSPSYYYYYGMDVW |
| HK_118 | #8 | IGHV4-39 | IGHJ5 | IGHD6-13 | unmutated | 21 | CARDTGYSSSWYGGGRWFDPW |
| HK_140 | #8 | IGHV4-39 | IGHJ5 | IGHD6-13 | unmutated | 21 | CARRVGYSSSWYDTYNWFDPW |
| HK_143 | #8 | IGHV4-39 | IGHJ5 | IGHD6-13 | unmutated | 21 | CASRAGYSSSWYSIPNWFDPW |
| NJ_22 | #8 | IGHV4-39 | IGHJ5 | IGHD6-13 | unmutated | 21 | CARRTGYSSSWYSNNNWFDPW |
| NJ_47 | #8 | IGHV4-39 | IGHJ5 | IGHD6-13 | unmutated | 21 | CARQVGYSSSWYDSYNWFDPW |
| NJ_48 | #8 | IGHV4-39 | IGHJ5 | IGHD6-13 | mutated | 20 | CAIQTGYSSSWYGVDWFDPW |
| NJ_160 | #8 | IGHV4-39 | IGHJ5 | IGHD6-13 | unmutated | 21 | CAARDAYSSSWYTGTNWFDPW |
| NJ_164 | #8 | IGHV4-39 | IGHJ5 | IGHD6-13 | unmutated | 20 | CARLTGYSSSWYSYNWFDPW |
| NJ_204 | #8 | IGHV4-39 | IGHJ5 | IGHD6-13 | unmutated | 21 | CASLDGYSSSWYQGVNWFDPW |
| NJ_353 | #8 | IGHV4-39 | IGHJ5 | IGHD6-13 | unmutated | 20 | CASRTGYSSSWYGDNWFDPW |
| NJ_03 | #8 | IGHV4-39 | IGHJ5 | IGHD6-13 | unmutated | 21 | CARRTGYSSSWYDRENWFDPW |
| NJ_412 | #8 | IGHV4-39 | IGHJ5 | IGHD6-13 | unmutated | 21 | CARRAGYSSSWYTTLNWFDPW |
| TJ_07 | #8 | IGHV4-39 | IGHJ5 | IGHD6-13 | mutated | 21 | CARTEGYSSSWYSTHNWFDPW |
| TJ_14 | #8 | IGHV4-39 | IGHJ5 | IGHD6-13 | unmutated | 20 | CATLRGYSSSWYGEDWFDPW |
| TJ_26 | #8 | IGHV4-39 | IGHJ5 | IGHD6-13 | unmutated | 20 | CATLRGYSSSWYGEDWFDPW |
| TJ_28 | #8 | IGHV4-39 | IGHJ5 | IGHD6-13 | unmutated | 20 | CAKRLGYSSSWYGVGWFDPW |
| TJ_59 | #8 | IGHV4-39 | IGHJ5 | IGHD6-13 | unmutated | 21 | CARMGGYSSSWYGSVNWFDPW |
| TJ_65 | #8 | IGHV4-39 | IGHJ5 | IGHD6-13 | mutated | 20 | CATLRGYSSSWYGEDWFDPW |
| HK_12 | #11 | IGHV3-7 | IGHJ4 | IGHD3-22 | mutated | 18 | CARGFYSGSYYANYFDSW |
| TJ_75 | #12 | IGHV1-18 | IGHJ4 | IGHD3-22 | unmutated | 21 | CARDPYYYDSSGYYFTRFDYW |
| TJ_115 | #13 | IGHV3-53 | IGHJ2 | IGHD2-21 | mutated | 21 | CARDRCSGKYCYRPDWHFDLW |
| NJ_373 | #16 | IGHV4-34 | IGHJ6 | IGHD2-15 | mutated | 26 | CASRFYCSGGGCQSPSYYYYYGMDVW |
| NJ_216 | #20 | IGHV3-66 | IGHJ4 | IGHD1-7 | mutated | 13 | CARGTTPGVFDYW |
| TJ_88 | #28A | IGHV7-4-1 | IGHJ6 | IGHD6-19 | unmutated | 19 | CARVYSSGWYYYYYGMDVW |
| TJ_119 | #28A | IGHV1-2 | IGHJ6 | IGHD1-26 | unmutated | 19 | CARLYSGSYYYYYYGMDVW |
| NJ_304 | #29 | IGHV4-34 | IGHJ3 | IGHD6-19 | mutated | 16 | CARDLAVPLPDAFDIW |
| NJ_350 | #29 | IGHV4-34 | IGHJ3 | IGHD6-19 | mutated | 16 | CARDLAVAPPDAFDFW |
| HK_147 | #31 | IGHV3-48 | IGHJ6 | IGHD2-2 | unmutated | 23 | CAREIGYQLLYMGDYYYYGMDVW |
| NJ_331 | #31 | IGHV3-48 | IGHJ6 | IGHD3-3 | unmutated | 23 | CARDPNFWSGYYSYYYYYGMDVW |
| HK_77 | #40 | IGHV4-39 | IGHJ4 | IGHD1-20 | mutated | 16 | CARATMTGTTGYFDYW |
| NJ_158 | #40 | IGHV2-5 | IGHJ5 | IGHD1-7 | unmutated | 15 | CAHSHITGTTGFDPW |
| HK_103 | #41 | IGHV1-69 | IGHJ6 | IGHD2-2 | unmutated | 25 | CARDGSDCSSTSCYEYYYYYYMDVW |
| NJ_201 | #41 | IGHV4-34 | IGHJ6 | IGHD2-2 | unmutated | 25 | CARQEAEYCSSTSCSPYYYYGMDVW |
| NJ_264_1 | #41 | IGHV4-34 | IGHJ6 | IGHD2-2 | unmutated | 25 | CARQEAEYCSSTSCSPYYYYGMDVW |
| HK_55 | #46 | IGHV3-9 | IGHJ4 | IGHD6-19 | mutated | 17 | CAKGAGYSIGWYYFDYW |
| NJ_286 | #50 | IGHV4-59 | IGHJ6 | IGHD3-22 | unmutated | 24 | CARGKGDYYNSSGYLVYYYGMDVW |
| HK_60 | #53 | IGHV3-53 | IGHJ4 | IGHD3-10 | mutated | 13 | CAKVDSGSLFDYW |
| TJ_111 | #55 | IGHV1-3 | IGHJ6 | IGHD3-10 | unmutated | 20 | CARFLWFGEPSYYYYGMDVW |
| TJ_157 | #57 | IGHV3-48 | IGHJ5 | IGHD3-3 | mutated | 20 | CAYSGRYYDFWSGYLGFDPW |
| HK_51 | #59 | IGHV1-58 | IGHJ5 | IGHD3-3 | unmutated | 14 | CAAGVDFWSGYPNW |
| HK_42 | #76 | IGHV3-23 | IGHJ4 | IGHD4-23 | mutated | 15 | CAKGDGGNSLPIDYW |
| HK_25 | #77 | IGHV4-59 | IGHJ5 | IGHD6-19 | mutated | 16 | CARGPNESGWLGLDQW |
| HK_61 | #77 | IGHV4-4 | IGHJ4 | IGHD6-19 | mutated | 16 | CARGADTTGWRPFDFW |
| TJ_29 | #77 | IGHV4-4 | IGHJ4 | IGHD5-12 | mutated | 16 | CARGPDYSGWNGFEYW |
| HK_49 | #89 | IGHV3-21 | IGHJ3 | IGHD3-22 | unmutated | 25 | CAREQYYYDSSGYLRSLRNDAFDIW |
| NJ_74 | #93 | IGHV3-48 | IGHJ5 | IGHD6-25 | unmutated | 11 | CARGTGVGDPW |
| NJ_362 | #99 | IGHV1-3 | IGHJ4 | IGHD6-19 | unmutated | 16 | CAREQWLVPNVNFDYW |
| TJ_73 | #99 | IGHV7-4-1 | IGHJ4 | IGHD6-19 | unmutated | 16 | CAREQWLVLVRHFDYW |
| NJ_191 | #234 | IGHV3-11 | IGHJ4 | IGHD3-10 | unmutated | 19 | CARVSLWFGELLRFDFDYW |
| TJ_18 | #234 | IGHV3-11 | IGHJ4 | IGHD3-10 | mutated | 19 | CARDLLWFGELLSYYFDYW |
| TJ_20 | #148B | IGHV2-5 | IGHJ4 | IGHD1-26 | mutated | 19 | CAHRHISGSPWDFGYFDYW |
| NJ_23 | #NEW1_CHINA | IGHV3-23 | 4IGHJ | IGHD2-21 | mutated | 20 | CAKGYRDNYDGDQSSVFDSW |
| NJ_186 | #NEW1_CHINA | IGHV3-23 | IGHJ4 | IGHD3-22 | mutated | 20 | CAKGYRDNYDGDQSSVFDSW |
| TJ_38 | #NEW10_CHINA | IGHV1-18 | IGHJ4 | IGHD6-6 | unmutated | 15 | CARLQYIPMYSLDYW |
| TJ_146 | #NEW10_CHINA | IGHV1-18 | IGHJ4 | IGHD6-13 | unmutated | 15 | CARLQYIPMYSLDYW |
| TJ_153 | #NEW11_CHINA | IGHV3-23 | IGHJ4 | IGHD2-2 | mutated | 10 | CVTGGQAGDW |
| TJ_156 | #NEW11_CHINA | IGHV3-23 | IGHJ4 | IGHD2-2 | mutated | 10 | CVTGGQAGDW |
| TJ_134 | #NEW12_CHINA | IGHV4-34 | IGHJ3 | IGHD6-19 | mutated | 17 | CARPYEVAVAPGAFDVW |
| TJ_136 | #NEW12_CHINA | IGHV4-34 | IGHJ3 | IGHD6-19 | mutated | 17 | CARPYEVAVAPGAFDVW |
| TJ_123 | #NEW13_CHINA | IGHV4-4 | IGHJ3 | IGHD5-12 | mutated | 20 | CARGASGYGLLGADDGFDVW |
| TJ_124 | #NEW13_CHINA | IGHV4-4 | IGHJ3 | IGHD5-12 | mutated | 20 | CARGASGYGLLGADDGFDVW |
| TJ_91 | #NEW14_CHINA | IGHV3-30 | IGHJ6 | IGHD5-12 | mutated | 13 | CARRGHDSYMDLW |
| TJ_150 | #NEW14_CHINA | IGHV3-30 | IGHJ6 | IGHD5-12 | mutated | 13 | CARRGHDSYMDVW |
| TJ_151 | #NEW14_CHINA | IGHV3-30 | IGHJ6 | IGHD5-12 | mutated | 13 | CARRGHDSYMDVW |
| TJ_56 | #NEW15_CHINA | IGHV3-74 | IGHJ4 | IGHD1-26 | mutated | 9 | CSWDHFDSW |
| TJ_143 | #NEW15_CHINA | IGHV3-74 | IGHJ4 | IGHD5-12 | mutated | 9 | CTYDHFDSW |
| NJ_78 | #NEW2_CHINA | IGHV4-59 | IGHJ4 | IGHD3-22 | unmutated | 22 | CARGAAGYYDSSGYKEYYFDYW |
| NJ_168 | #NEW2_CHINA | IGHV4-59 | IGHJ4 | IGHD3-22 | unmutated | 22 | CARRGSGYYDSSGYPEYYLDYW |
| NJ_254 | #NEW3_CHINA | IGHV3-21 | IGHJ4 | IGHD3-22 | unmutated | 21 | CARESYYYDISGYFLYYFDYW |
| NJ_266 | #NEW3_CHINA | IGHV3-21 | IGHJ4 | IGHD3-22 | unmutated | 21 | CARESYYYDISGYFLYYFDYW |
| NJ_136 | #NEW4_CHINA | IGHV3-74 | IGHJ3 | IGHD6-19 | unmutated | 14 | CARAVAGIDAFDIW |
| NJ_42 | #NEW4_CHINA | IGHV3-74 | IGHJ3 | IGHD6-19 | unmutated | 14 | CARAVAGIDAFDIW |
| HK_85 | #NEW5_CHINA | IGHV4-4 | IGHJ4 | IGHD3-22 | unmutated | 18 | CARGGGYSSGYPYYFDYW |
| NJ_300 | #NEW5_CHINA | IGHV4-4 | IGHJ4 | IGHD6-19 | unmutated | 18 | CARGGGYSSGLPYYFDYW |
| HK_19 | #NEW6_CHINA | IGHV3-7 | IGHJ6 | IGHD3-10 | unmutated | 20 | CAAGRGSGRYYYYYYGMDVW |
| NJ_71 | #NEW6_CHINA | IGHV3-7 | IGHJ6 | IGHD1-26 | unmutated | 20 | CAGGSGSYQYYYYYYGMDVW |
| TJ_03 | #NEW7_CHINA | IGHV3-9 | IGHJ6 | IGHD6-13 | mutated | 24 | CAKDRVCCIAAAGPSLRYYGMDVW |
| TJ_155 | #NEW7_CHINA | IGHV3-9 | IGHJ6 | IGHD6-13 | mutated | 24 | CAKDRVCCIAAAGPSLRYYGMDVW |
| TJ_27 | #NEW8_CHINA | IGHV3-23 | IGHJ4 | IGHD3-16 | mutated | 29 | CAKDVRGGREEFADYIWVSYRSGIAFDYW |
| TJ_45 | #NEW8_CHINA | IGHV3-23 | IGHJ4 | IGHD3-16 | mutated | 29 | CAKDVRGGREEFADYIWVSYRSGIAFDYW |
| TJ_32 | #NEW9_CHINA | IGHV4-34 | IGHJ5 | IGHD2-21 | mutated | 21 | CARRRDPVYCGDDCPTGVDSW |
| TJ_34 | #NEW9_CHINA | IGHV4-34 | IGHJ5 | IGHD2-21 | mutated | 21 | CARRRDPVYCGDDCPTGVDSW |
| NJ_15 | #NOVEL 1 | IGHV4-59 | IGHJ6 | IGHD3-22 | unmutated | 25 | CARGNYYDSSGYYYVGYYYYYMDVW |
| NJ_31 | #NOVEL 1 | IGHV4-59 | IGHJ6 | IGHD3-22 | unmutated | 25 | CARGDYYDSSGYYYVGYYYYYMDVW |
